# Effects of point mutations in the binding pocket of the mouse major urinary protein MUP20 on ligand affinity and specificity

**DOI:** 10.1038/s41598-018-36391-3

**Published:** 2019-01-22

**Authors:** Jimena Ricatti, Laura Acquasaliente, Giovanni Ribaudo, Vincenzo De Filippis, Marino Bellini, Ramiro Esteban Llovera, Susi Barollo, Raffaele Pezzani, Giuseppe Zagotto, Krishna C. Persaud, Carla Mucignat-Caretta

**Affiliations:** 1 Department of Molecular Medicine, University of Padua, Padua, Italy; 2 Cell Biology and Neuroscience Institute, University of Buenos Aires—National Scientific and Technical Council (UBA-CONICET), Buenos Aires, Argentina; 3 Department of Pharmaceutical and Pharmacological Sciences, University of Padua, Padua, Italy; 4 Multidisciplinary Institute of Cell Biology, National Scientific and Technical Council (CONICET) and Department of Science and Technology, National University of Quilmes, Buenos Aires, Argentina; 5 Department of Medicine, University of Padua, Padua, Italy; 6 School of Chemical Engineering and Analytical Science, University of Manchester, Manchester, UK; 7 National Institute of Biostructures and Biosystems, Rome, Italy

## Abstract

The mouse Major Urinary Proteins (MUPs) contain a conserved β-barrel structure with a characteristic central hydrophobic pocket that binds a variety of volatile compounds. After release of urine, these molecules are slowly emitted in the environment where they play an important role in chemical communication. MUPs are highly polymorphic and conformationally stable. They may be of interest in the construction of biosensor arrays capable of detection of a broad range of analytes. In this work, 14 critical amino acids in the binding pocket involved in ligand interactions were identified in MUP20 using *in silico* techniques and 7 MUP20 mutants were synthesised and characterised to produce a set of proteins with diverse ligand binding profiles to structurally different ligands. A single amino acid substitution in the binding pocket can dramatically change the MUPs binding affinity and ligand specificity. These results have great potential for the design of new biosensor and gas-sensor recognition elements.

## Introduction

**Figure 1:**
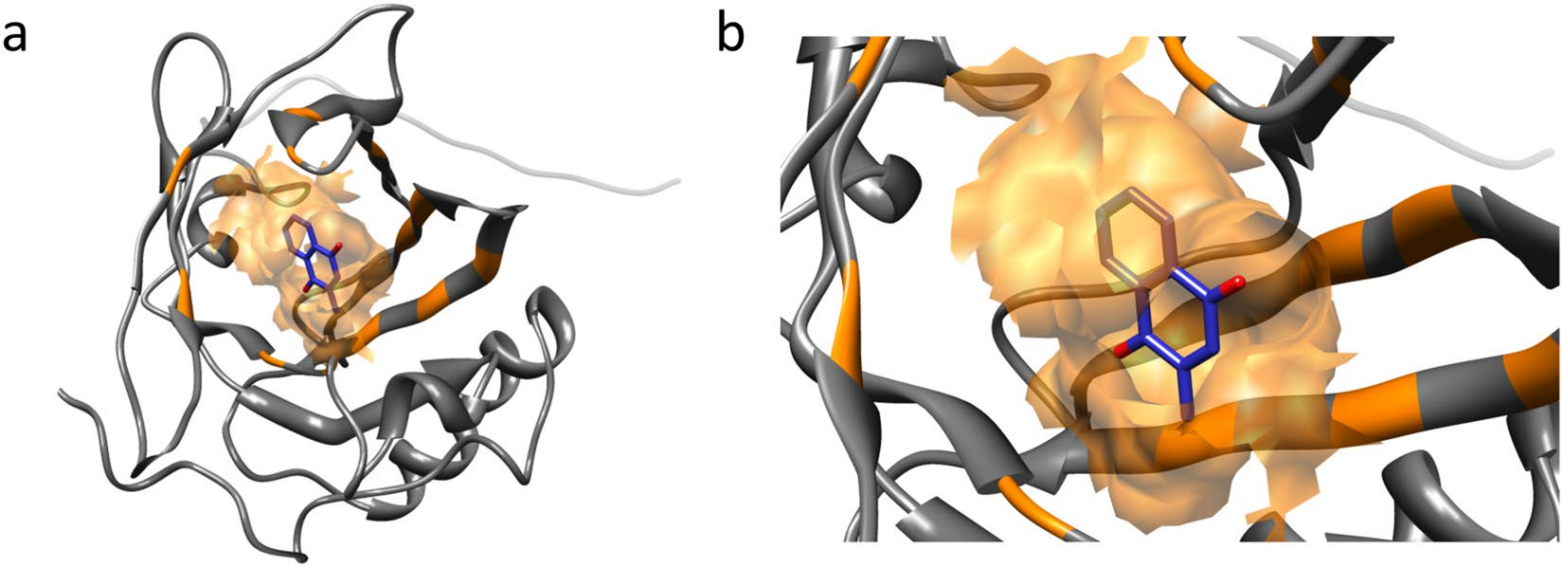
Three-dimensional structure of murine MUP20 with menadione inside the binding cavity. (**a**) Inside the binding pocket, the ligand structure is depicted in blue with oxygen in red; the binding cavity surface and the interacting residues are highlighted in orange. The ligand is buried within the binding cavity and appears partially covered by the binding surface. (**b**) A closer view of the binding pocket reveals that the aromatic ring and the methyl group of menadione are inside the cavity. Docking was performed with Autodock/Vina^42,43^, starting from the first conformer of the NMR structure of MUP20 (2L9C.pdb).

Major urinary proteins (MUPs) in the mouse belong to the lipocalin family and contain a conserved β-barrel structure with a characteristic central hydrophobic pocket^1,2^. They are mainly synthesised in the liver and excreted into the urine^3^. The tertiary structure of MUPs consists of eight β-strands, arranged in an anti-parallel β-barrel open on one side, with α-helices at both ends, encompassing a cup-like binding pocket (Fig. 1). They bind volatile pheromones or other lipophilic molecules^4,5^, are transported across the kidney, and excreted in the urine^6^. Mice deliberately deposit numerous small urine drops, that act as scent marks. Once deposited in urine marks, MUPs release bound volatile compounds slowly into the air. These are highly important for chemical communication^7,8^ and indeed mice recognise scent marks by their molecular composition^9^.

The stability of lipocalins, like vertebrate Odorant Binding Proteins (OBP), against proteolysis, temperature, and solvents make them attractive for development of biosensors^10^. Recent work has shown that OBPs can be successfully integrated into a variety of transducers, including quartz crystal microbalances, surface acoustic wave devices, organic field effect transistors^11–15^, to produce gas sensors. If the structures of the binding sites can be tailored to produce proteins capable of sensitive and selective detection of ligands for specific applications, then this would open a new generation of gas sensing technology. MUPs are interesting candidates for this purpose. They are highly polymorphic, and the relative abundance of different isoforms in urine is thought to function as individual identity signatures of mice^16,17^. However, the relationship between extreme polymorphism and chemical communication is not clear^18^. There are 21 MUPs isoforms characterized by small differences in amino acid sequences, highly conserved biochemical properties and tertiary folding features. The sites of variation are found only on a restricted segment of the polypeptide chain, which projects to a patch on the surface of the protein^16^. Each isoform displays a unique spectrum of binding specificities across a variety of ligands^19^. These proteins and their volatile ligands are detected by the main and accessory olfactory systems of the mouse and trigger adaptive behavioural responses and/or physiological processes^8,20^. Apart from the release of volatiles into the environment, there is evidence that the proteins themselves may also function as chemosignalling molecules^21–23^, supporting the segregation of the Mus genus species^24^.

Of the MUP proteins, MUP20 is also known as Darcin for its peculiar expression in male mice and for its primary role in social communication^25,26^, but there is limited information available about the critical amino acid residues of the binding pocket involved in interactions with the ligand. We utilised this protein for our work. The approach taken here was to use known structures of MUPs to identify the critical amino acids in the binding pocket that interact with ligands. A subset of possible mutant proteins was identified, that could form an array of proteins with diverse ligand binding characteristics. From these studies, an interesting outlook of artificial sensing systems for biotechnological applications of MUPs is now emerging^10,27^.

## Results

### Selection of MUP20 ligands

While the lock-and-key model may be inadequate, bioisosteric substitutions may be relevant for exploring odorant-protein interactions^28^. Here, six chemically unrelated ligands, some of which display odorant/pheromone characteristics^9,25,29^, were selected for their different physico-chemical properties, i.e. molecular size, chemical structure, hydrophilic/hydrophobic balance, and hydrogen bonding properties (Table 1). In particular, 2-isobutyl-3-methoxypyrazine (PYR) is a low-threshold odor^30,31^, used in MUPs/ligand binding studies^4,7^, methylnapthalene-1,4-dione (menadione, MEN), a naphtoquinone analogue of vitamin K is also found in MUP scent marks^32^ while 2,4-dimethylphenol (2,4-DMP), and 2-butyl-1-octanol (OCT), along with linalool (3,7-dimethylocta-1,6-dien-3-ol) (LIN), contribute to urine scent marks in male mice^9,33^. L-Adrenaline (L-ADR) was also tested as a precursor of vanilloid ligands, which may bind to Transient Receptor Potential Vanilloid (TRPV) receptors widely found in the olfactory epithelium^34^.

**Table 1:** List of ligands used for docking simulations with MUP20 and their physico-chemical properties. The molecular mass values, the molecular volumes and the octanol/water partition coefficients (LogP) are reported along with the ZINC accession.

Physico-chemical properties of selected MUP ligands.				
Ligand	Molecular mass (a.m.u.)	Molecular Volume (Å^3^)^a^	LogP^a^	ZINC Accession Number^b^
L-Adrenaline (L-ADR)	183.20	171	−0.82	ZINC00039089
Methylnapthalene-1,4-dione (Menadione, MEN)	172.18	155	1.91	ZINC00001677
2,4-Dimethylphenol (2,4-DMP)	122.16	125	2.37	ZINC01672873
2-Butyl-1-octanol (OCT)	186.33	222	5.36	ZINC1640892
Linalool (LIN)	154.25	176	2.68	ZINC01529819
2-Isobutyl-3-methoxypyrazine (PYR)	166.22	168	2.11	ZINC00156517

^a^Values of molecular volume and LogP were determined using the online Molinspiration software version 2011.06 (www.molinspiration.com) and ALGOPS2.1 program^71^.

^b^Accession number of the listed molecules in the ZINC data base^69^.

### Selection of MUP20 mutations

**Figure 2:**
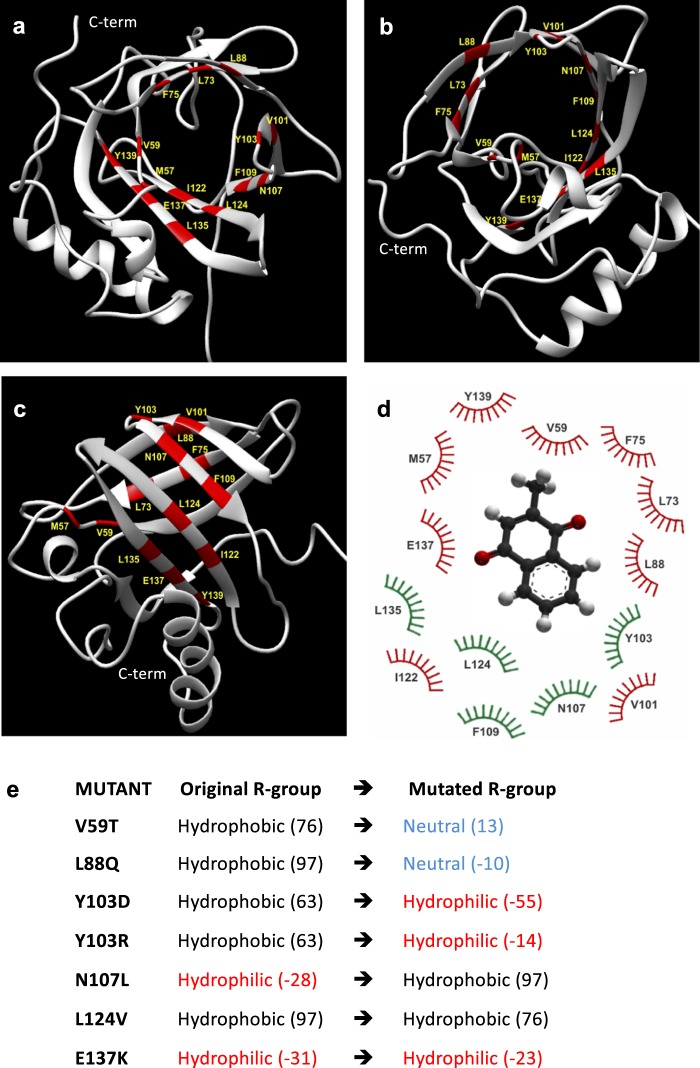
(**a**–**c**) The structure of wild type MUP20 (PDB ID: 2L9C, 6xHis tag is not represented) in different orientations. The 3D representation shows in red the amino acids belonging to the hydrophobic pocket involved in the binding process, according to the LPC/CSU analysis. C-term indicates C-terminus. (**d**) Relevant residues involved in the ligand-MUP20 interaction. Those in green are conserved across the natural MUP isoforms, while the variable residues are in red. (**e**) Amino acid mutations in MUP20. The polar/apolar nature of the original and substituted amino acids is reported together with the corresponding hydrophobicity index values (in brackets)^36^.

Figure 1 illustrates the tertiary structure of MUP20 (PDB ID: 2L9C.pdb). The ligand-binding pocket in MUP20 is shaped by a region containing mostly hydrophobic amino acids (1 Ile, 2 Phe, 4 Leu, 2 Val, and 1 Met) lining a cavity of approximately 435 Å^3^ (Fig. 2a–c). Interestingly, the polar –OH groups of two Tyr side-chains (103 and 139) protrude into the apolar cavity surface of MUP20, along with the hydrophilic side chain of Asn107 and the negatively charged Glu137, which in the crystallographic structure of the sequence/structural homologous MUP4 (PDB ID: 3KFF) is partially compensated by two structural water molecules^35^.

From the computational analysis of interatomic contacts on digital models of MUP20 mutants docked with menadione, a reference ligand for MUPs^32^, fourteen amino acids localised in the binding pocket were identified as the most relevant for interactions with ligands (Table 2, Fig. 2d). Notably, the residues shown in green are conserved across all natural MUP isoforms. These were ranked on the basis of minimum distance existing between the amino acids in the binding pocket and the ligands, as well as according to the greater contact surface. The calculated ligand-MUP distances obtained from the LPC/CSU analysis ranged from 2.5 to 5.5 Å, while the contact surface area was estimated in the 5.8–46.3 Å^2^ range (Table 2). From these data, six critical residues in the binding region were selected for substitution in order to produce an effective mutation that would change the binding characteristics of MUP20 (Fig. 2e), while retaining the structural stability of the protein. The selected amino acids included strictly conserved residues in MUP20, like Tyr103, Asn107, and Leu124, as well as partially conserved residues, like Val59, Leu88, and Glu137 (Fig. 2d). The substituent amino acids were selected according to their size and hydrophobicity index^36^ and mutations were designed to mainly alter the hydrophilic/hydrophobic balance at the mutation site, without significantly perturbing the conformational and stability properties of MUP20 (see Supplementary S1 for 3D models and Root-mean-square deviation of atomic positions, RMSD, data).

**Table 2:** LPC/CSU analysis of the interactions of the amino acids in the ligand-binding pocket of MUP20 with menadione.

Residue position	Distance^a^	Surface^b^	Hphi-Hphi^c^	A-A^d^	Hpho-Hpho^e^	Hpho-Hphi^f^
M57	5.5 Å	11.7 Å^2^	−	−	**+**	−
V59	4.8 Å	24.0 Å^2^	−	−	**+**	−
L73	3.3 Å	36.8 Å^2^	−	−	**+**	−
F75	5.0 Å	19.1 Å^2^	−	−	**+**	−
L88	3.6 Å	26.7 Å^2^	−	−	**+**	−
V101	4.9 Å	7.2 Å^2^	−	−	**+**	−
Y103	3.4 Å	41.5 Å^2^	−	−	**+**	−
N107	4.7 Å	5.8 Å^2^	−	−	−	**+**
F109	3.7 Å	35.9 Å^2^	−	−	**+**	−
I122	3.9 Å	22.2 Å^2^	−	−	**+**	−
L124	3.4 Å	20.3 Å^2^	−	−	**+**	**+**
L135	3.8 Å	13.0 Å^2^	−	−	**+**	−
E137	2.5 Å	46.3 Å^2^	**+**	−	−	**+**
Y139	3.4 Å	14.3 Å^2^	**+**	−	−	−

^a^The closest measured distance (Å) between atoms of the ligand and the residue. ^b^Contact surface area (Å^2^) between the ligand and the residue. ^c^Hydrophilic-hydrophilic contact. ^d^Aromatic-aromatic contact. ^e^Hydrophobic-hydrophobic contact. ^f^Hydrophobic-hydrophilic contact.

The amino acid substitutions shown in Fig. 2e were also intended to exaggerate the effect of variations found in natural MUP, within the constraints of also maintaining conformational stability of the protein. Although V59 is not strictly conserved in MUP family, possibly replaced by more hydrophobic or less hydrophobic amino acids like Leu and Ala, respectively, the naturally occurring residues at position 59 are all hydrophobic in nature. Hence, we decided to perturb position 59 by replacing Val with Thr, which retains the conformational properties of Val, whereby both residues are strong β-sheet stabilizers. However, due to the presence of the –OH group, Thr is much less hydrophobic than Val (see Table 1)^36^. At position 88, Leu, Ala or Met are observed in natural MUP isoforms and even in this case all these residues are highly hydrophobic. Here Leu88 was replaced by Gln, which has a flexible and polar side chain amenable to form stable hydrogen bonds. At position 137 Glu and Gly residues are frequently observed in MUP family, where Glu has a negatively charged and flexible side-chain while Gly is neutral and dramatically increases the conformational flexibility of the backbone^37^. Here, we decided to alter the charge and conformational properties at position 137 by replacing Glu with Lys, which has a long/flexible and positively charged side chain. Among the naturally conserved amino acids, Leu124 was replaced with Val, where Val-side chain retains the apolar character of Leu but has a smaller size and different conformational properties. Actually, Leu is one of the best helix-stabilizers among the natural amino acids, whereas Val is a helix-breaker and stabilizes the β-sheet secondary structure. Next, drastic chemical variations were introduced at position 103, where the rigid aromatic side-chain of the Tyr-residue in wild-type MUP20 was replaced by Arg, having a long and flexible positively charged side chain. To test the role of the electrostatic charge at position 103, Tyr103 was also replaced by Asp, which is negatively charged. Finally, the neutral and polar side chain of Asn107 was substituted with the apolar side chain of Leu.

The selected ligands have different chemical features such as steric hindrance and hydrophilic/hydrophobic properties (see Table 1).

**Figure 3:**
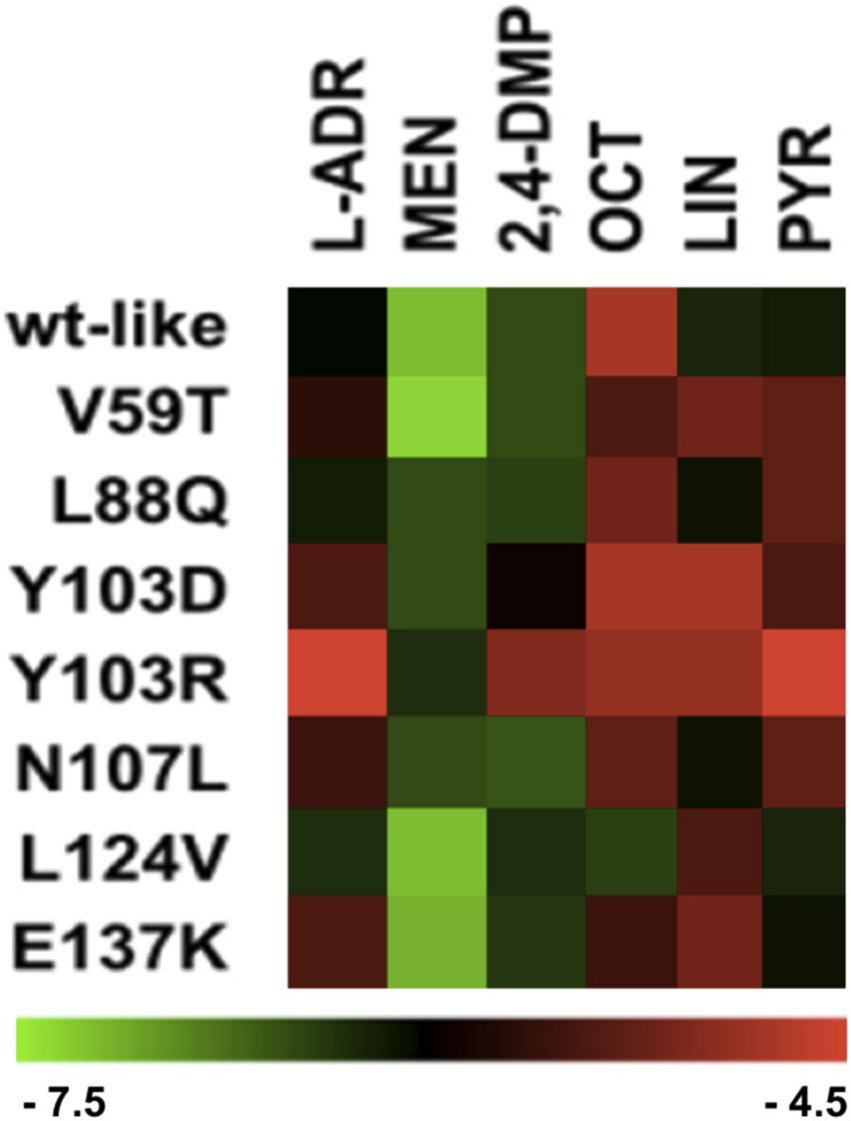
*In silico* binding analysis. Theoretical binding energies (ΔG_b_) (kcal/mol) of the selected ligands to wild type (WT) MUP20 and mutant models, plotted as a heatmap. The change of ΔG_b_ values across mutants, and between different ligands are represented from light green to light red. Refer to the Supplementary File S1 for ΔG_b_ values.

The theoretical binding profiles of these ligands to the seven selected MUP20 mutants are depicted in Fig. 3. Predicted ligand affinities to MUP mutants are expressed as theoretical free energy change of binding (ΔG_b_)^38^ and are the result of blind docking simulations. Accordingly, the wild type MUP20 (WT) displayed binding selectivity for MEN and to a lesser extent for 2,4-DMP and this binding profile was roughly retained for V59T, L88Q, L124V, N107L and, rather surprisingly, even for the mutant carrying the charge reversal E137K. The mutant L124V showed the broadest specificity profile, compared to other mutants, whereas Y103R and Y103D displayed a strong reduction in the ability to recognize the selected ligands, such that these mutants were predicted to bind solely to MEN. According to our *in silico* experiments, while most of the ligands cluster in the internal MUP20 pocket, a limited number of interacting molecules may also bind to an external portion of the protein in some of the models (see Supplementary File S1 for complete 2D interaction patterns).

### Production, purification and characterization of MUP20 mutants

**Figure 4:**
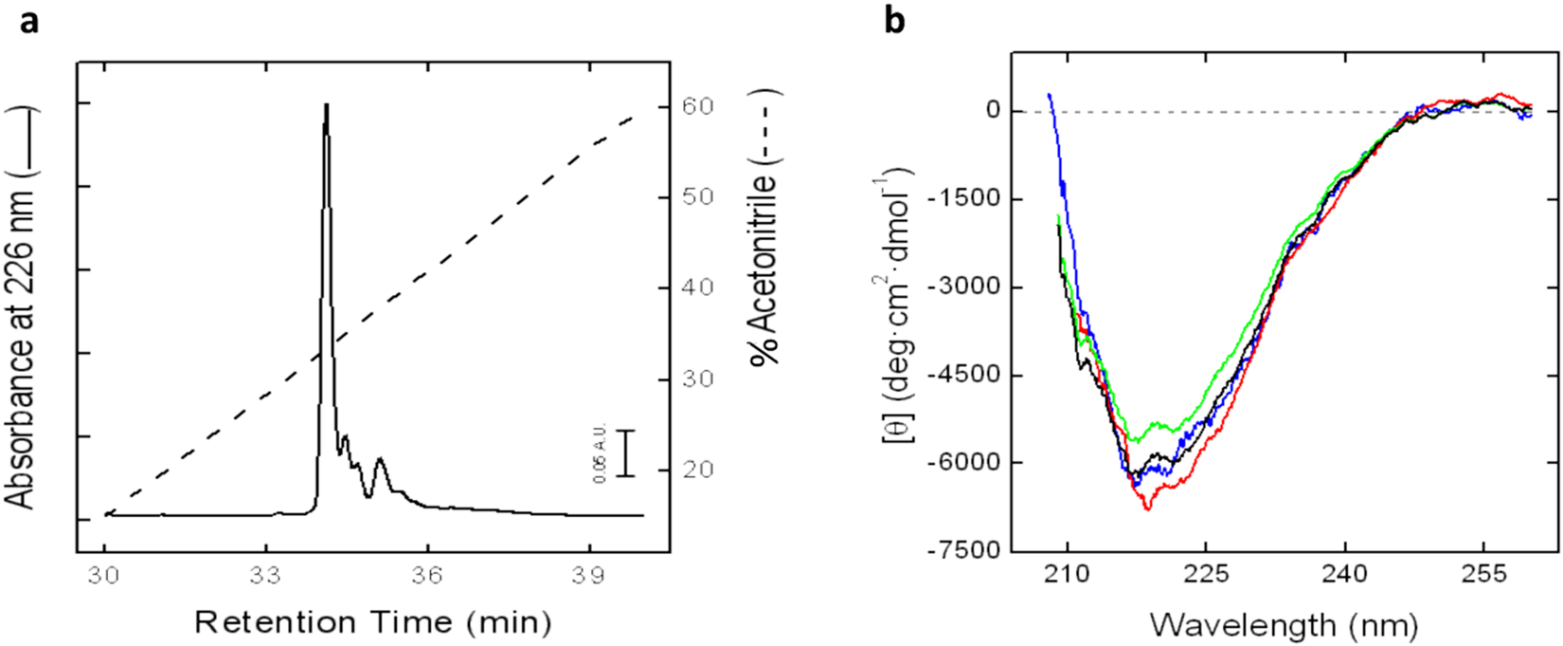
Characterization of recombinant MUP20 proteins. (**a**) RP-HPLC analysis of recombinant wild type MUP. An aliquot (20 μg) of protein was applied to a Vydac C4 analytical column eluted with a linear acetonitrile-0.1% TFA gradient (—) at a flow rate of 0.8 ml/min. The protein material corresponding to the major peak in the chromatograms was collected and subjected to MS analysis (Supplementary Material S3). (**b**) Far-UV CD spectra of wild-type and mutant MUPs. CD spectra of recombinant wild type MUP (black line), and MUP mutants: L88Q (blue line), L124V (red line) and Y103R (green line). All measurements were carried out at 25 ± 0.1 °C 10 mM Na_2_HPO_4_ pH 7.4, 0.15 M NaCl, at a protein concentration of 0.1 mg/ml.

To test the working hypotheses that emerged from the *in silico* analysis, wild type MUP20 and the seven selected MUP20 mutants were expressed in *E*. *coli* as 6xHis-tagged N-terminal fusion proteins, as detailed in the Methods section, and further characterized with respect to their chemical identity, conformational and ligand-binding properties. Recombinant MUPs were purified to homogeneity by IMAC, as documented by reducing SDS-PAGE and Coomassie staining, where MUP proteins migrated as a single band (>85%) with an apparent molecular weight of ~20 kDa (Supplementary Fig. S2b). The homogeneity of recombinant MUPs preparations was further checked by RP-HPLC (Fig. 4a), while high-resolution mass spectrometry analyses yielded mass values in agreement with those deduced from the amino acid composition of MUP analogues within 50 ppm mass accuracy (Supplementary Material S3), thus providing clear-cut evidence for the chemical identity of mutant MUPs. To verify that point mutations did not appreciably affect the conformation of MUP20, circular dichroism CD spectra were recorded in the far-UV region (Fig. 4b), a spectroscopic technique that provides key information on the protein secondary structure content. The CD spectrum of recombinant wild-type MUP20 displays a single minimum centered at about 218 nm, reflecting the high β-sheet content of MUP family members^1^. Notably, the spectra of MUP20 mutants are almost superimposable to that of the wild-type protein, thus suggesting that the single point mutations inserted into MUP20 sequence do not alter the overall MUP structure. Importantly, the latter result allows us to interpret ligand binding data solely on the basis of the different physico-chemical properties of the ligands and/or of the mutated amino acid side chain.

### Fluorescence and calorimetric ligand binding measurements to MUP20 mutants

**Figure 5:**
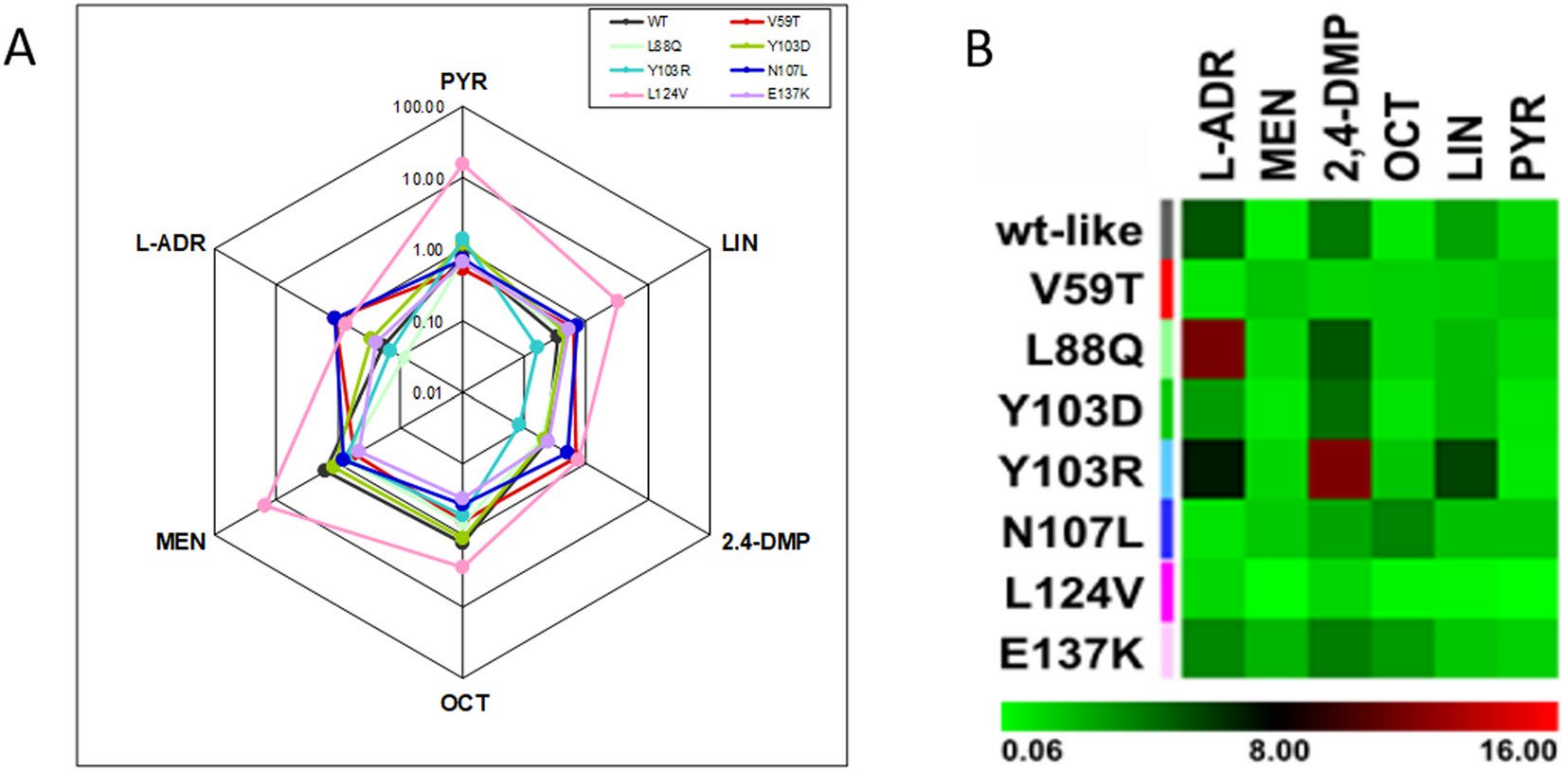
*In vitro* binding analysis. (**A**) Radar plot of the binding profile for displacement of the fluorescent ligand 1-NPN from MUP mutants. The equilibrium association constants, K_a_, relative to the binding of each ligand to each mutant MUP are plotted on a logarithmic scale. (**B**) The equilibrium dissociation constants, K_d_, are plotted as a heatmap, displaying the variations across mutants, and between different ligands binding to the same mutant. The change of K_d_ values are represented from light green to light red and the numbers refer to the range of dissociation constants (µM).

The affinity of the six ligands reported in Table 1 for wild-type and seven mutant MUPs was first determined by fluorescence competition experiments, using 1-NPN as a fluorescent MUP ligand^39,40^. Using this method, rough estimates of ligand affinity for MUPs were obtained and graphically represented in Fig. 5 as radar plot and heatmap. Wild-type MUP20 binds to the selected ligands with a K_d_ range of 0.64 to 5.36 μM, with the highest affinity to menadione, followed by PYR and OCT (Supplementary Materials S4 and S5). The heatmap indicates that each mutant had a different selectivity profile, with MUP20 L124V showing less specificity in binding to all the selected ligands, and Y103R showing the highest selectivity. Intriguingly, N107L mutant displayed a binding profile remarkably different from that of the wild-type MUP20.

**Figure 6:**
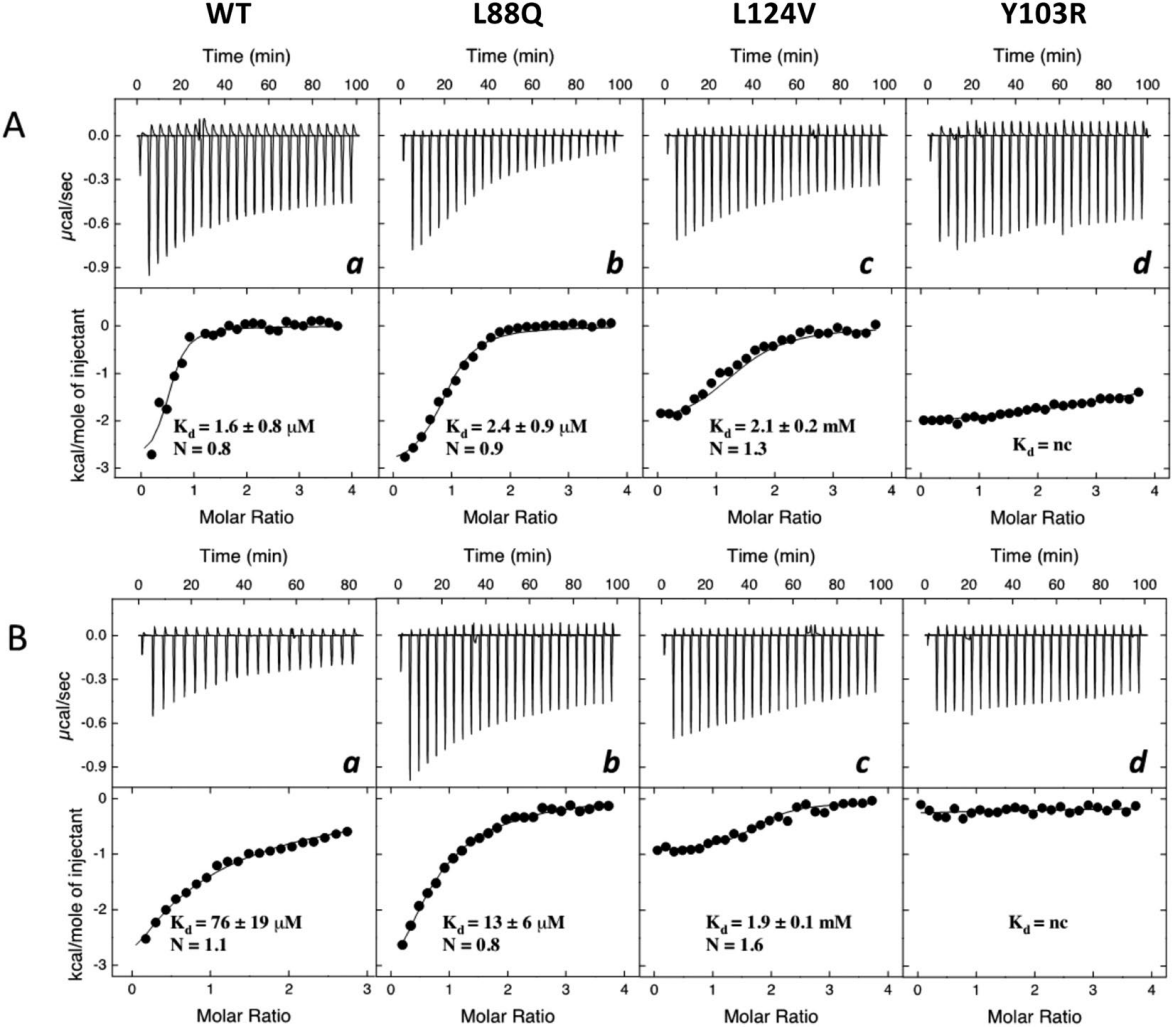
ITC binding measurements. Binding of 2-isobutyl-3-methoxypyrazine (PYR) and menadione (MEN) to wild type and mutant MUPs. ITC data for PYR (**A**) and MEN (**B**) binding to recombinant wild type MUP (WT) (a), and mutants L88Q (b), L124V (c) and Y103R (d). Raw injection heats (expressed as differential power) are shown in the top panels and the corresponding specific binding isotherms, calculated from the integrated injection heats and normalized to the moles of injectant, are shown in the bottom panels. The equilibrium dissociation constants (K_d_) and stoichiometry values (N) were determined by fitting the data to a one-site binding model.

The binding energetics of the best ligands, previously identified in fluorescence binding measurements (see Fig. 5), i.e. 2-isobutyl-3-methoxypyrazine (PYR) and menadione (MEN), to wild-type (WT) and seven mutant MUPs was studied by isothermal titration calorimetry (ITC). This gave an independent analysis of binding without the constraints of ligand displacement experiments, i.e. linearity of fluorescence changes and possible unspecific interactions with 1-NPN^39^. The titration of wild type MUP20 and mutants L88Q and L124V gave an exothermic reaction with both PYR and MEN (Fig. 6A,B), approaching saturation at high ligand/MUP molar ratio, yielding a ligand:MUP binding stoichiometry close to 1. Notably, the binding strength of the same ligands to Y103R mutant (Fig. 6A,B panel d) was barely detectable.

ITC measurements indicate that PYR binds to the wild type MUP20 with an affinity >40 fold higher than MEN: this may be due to the nature of the pyrazine ring of PYR which can form more hydrogen bonds than the naphtoquinone scaffold of MEN, when inserted into the binding pocket of MUP20. The replacement of L88 with a linear and more polar amino acid such as Gln did not appreciably affect the affinity for PYR, whereas the same L88Q mutation increased the affinity for MEN by about 6-fold. On the other hand, the mutation of L124 with the smaller and hydrophobic valine residue resulted in a drop of the affinity for PYR and MEN by 1300- and 25-fold, respectively, compared to the wild-type MUP20. Notably, the L124V mutant displayed identical affinity, within the limits of the experimental error, for both PYR and MEN.

## Discussion

The phylogenetic framework of chemical communication of mice is complex. Different MUP isoforms may trigger a variety of behavioural and physiological effects. MUP bind a variety of small molecules^7,41^ which may be used for communication among mice, suggesting that they may bind other molecules of interest. However, it is still not clear how variations in amino acid sequence across isoforms that are minimal in many cases, may affect binding of ligands or their release to the environment. Here, we establish the basis for understanding how a substitution of one critical amino acid in the binding pocket can produce a large change in selectivity to volatile ligands that may be bound, as well as their affinities.

In this study, we used the solution structure of MUP20 to digitally dock MEN, as a prototype of MUP ligands (Fig. 1), and identify the potential hot spots on MUP20 structure important for ligand binding. The changes in binding affinity of WT and mutant MUPs for the six chemically unrelated ligands were first estimated by docking simulations, expressed as free energy change of binding (ΔG_b_) (Fig. 3). Starting from the results of simulations, seven point-mutants of MUP20 were expressed, purified and thoroughly characterized with respect to their purity, chemical identity, and conformational properties. Thereafter, experimental values of the dissociation constants (K_d_) of each mutant MUP for the selected ligands were determined by fluorescence competition measurements using 1-NPN as a fluorescent MUP ligand (Fig. 5). The complex figure that emerges from the affinity data is that each mutant MUP has a different binding selectivity profile and that a given mutation can perturb binding of a specific ligand without substantially affecting the affinity of the others. The comparison of the theoretical and experimental binding data (Figs 3 and 5) highlights the partial predictive power of our theoretical approach. The docking protocol used in this study assumes that (i) ligand-receptor interaction follows a rigid-body mechanism, (ii) the contribution of desolvation is negligible, and (iii) the receptor structure remains unchanged upon mutation^42,43^. These assumptions can hardly apply to ligand-MUP binding: the high conformational flexibility of MUPs, their structural plasticity in ligand binding, and the key role that solvation water molecules play in molecular recognition of MUPs are all implicated in the structure-function relationship^44–50^. All these contributions make difficult to accurately predict the effect of mutations in MUP binding. Notwithstanding, docking simulations have been a useful tool in guiding the selection of potential mutants for expression.

Considered the complex nature of ligand-MUP interaction and the moderate/low expression yields of mutant MUPs, we used ITC to measure the affinity of the best ligands identified in competition experiments (i.e. MEN and PYR) to WT and some selected mutant MUPs (i.e. L88Q, Y103R, and L124V), being ITC the gold standard technique for studying ligand-receptor interaction in a label-free system^51^. From the thermograms in Fig. 6 it was possible to extract K_d_ values for the binding of PYR to WT (K_d_ = 1.6 μM) and L88Q (2.4 μM), which were in excellent agreement with the affinities deduced from fluorescence competition experiments, yielding K_d_(WT-PYR) = 1.3 μM and K_d_(L88Q-PYR) = 1.4 μM. ITC data, however, indicate that the replacement of Leu124 with Val leads to a drop in the affinity for both PYR and MEN. Likewise, the substitution of the aromatic Tyr103 with the long, positively charged Arg-residue almost abolished binding of both ligands tested. Intriguingly, fluorescence measurements were able to pinpoint this dramatic drop in the affinity of Y103R mutant only for the binding to L-ADR, 2,4-DMP, and LIN. These apparent discrepancies between fluorescence and ITC measurements can be reconciled considering that, contrary to ITC which directly measures the heat exchange of ligand binding, the spectroscopic method used here to estimate ligand-MUP affinities is actually an indirect procedure relying on the decrease of the fluorescence intensity upon displacement of the MUP-bound fluorophore 1-NPN, when increasing concentrations of a competing ligand in the solution. Noteworthy, the results of competition binding measurements are affected by poor linearity of the spectroscopic signal, possibly caused by fluorescence inner filter effects^52^, along with the unique binding selectivity profile of 1-NPN for the mutant MUPs. Furthermore, an increase (instead of a decrease) of fluorescence intensity may be observed with some ligands, as a result of unpredictable unspecific interaction of the added ligand with the apolar 1-NPN fluorophore either in the free or MUP-bound state^39^.

Beyond comparing the accuracy of fluorescence and calorimetric techniques, the data in Fig. 6 indicate that position 88 in MUP20 structure is quite tolerant to mutation, as the substitution of the apolar Leu with the polar Gln-residue does not alter the affinity of L88Q for PYR and even improved the binding strength for MEN by 6-fold compared to WT. Leu88 is not conserved across the MUP family members (Fig. 2d). Therefore, our affinity data are consistent with the general trend that mutations at evolutionary non-conserved positions usually do not significantly alter neither protein structure nor function, whereas even subtle changes at conserved sites dramatically affect structure, stability and function^53^. Hence, the replacement of the highly conserved Leu124 with Val dramatically decreased the affinity for both the ligands tested. The lack of the methylene-group, upon Leu → Val exchange, may alter the physico-chemical forces driving ligand-MUP interaction and may stabilize the apolar pocked of MUP20 into a conformation unproductive for ligand binding. This interpretation is realistic considering that the energetics of ligand-MUP interaction is intrinsically complex and contributed by several different interactions, including hydrophobic desolvation of both the protein and the ligand, formation of a buried water-mediated hydrogen bond network between the protein and ligand, formation of strong van der Waals interactions, and changes in the structure, dynamics, and/or hydration of the protein upon binding^44–50^. Moreover, ligand-MUP interactions follow an induced-fit mechanism, whereby the apolar binding cavity is more occluded in the ligand-free protein than in the ligand-bound structure^2^. The interaction pocket in MUPs is optimised for a variety of ligand binding modes, thus accounting for the known promiscuity of MUPs in ligand binding^54^. A further complication in ligand-MUP interaction stems from NMR data showing that, counterintuitively, the backbone flexibility of MUP slightly increases upon ligand binding^55^. The combination of opposing entropic and enthalpic effects make difficult to interpret binding data to MUPs on rigorous structural grounds. In the case of Leu124Val mutation, it is likely that even a small reduction in the amino acid side-chain volume of Leu124 (vol = 124 Å^3^), after mutation with Val (vol = 105 Å^3^), creates an uncompensated cavity in the binding pocket of MUP20 that induces a rearrangement of the surrounding amino acids to a conformation that disfavours ligand binding. Actually, far-UV circular dichroism data indicate that all the mutant MUPs retain the overall secondary structure of the wild type MUP20 (Fig. 4b). Nevertheless, there may be subtle but functionally important changes in the local structure and dynamics of the binding pocket which cannot be detected by far-UV CD measurements^56^. This would indicate that there is great scope for MUP analogues to have a different binding selectivity profile depending on small, elusive structural changes in the apolar cavity. Lastly, the replacement of the conserved Tyr103 with Arg at the entrance of the binding cavity almost abrogates affinity for the ligand molecules tested (Fig. 6). Previous work from different laboratories have shown that the presence of an aromatic amino acid, like Tyr and Phe, is required to stabilize the binding of natural ligands to MUPs and other lipocalins important for chemical communication in mice and rats^32,57^ and also in other lipocalins like the Odorant Binding Proteins^58^. The dramatic effect of Tyr → Arg exchange might be expected considering the drastic structural change associated to the mutation of the aromatic and rigid Tyr-residue with the long/flexible and positive Arg side chain, protruding into and limiting the access to the apolar cavity of MUP20.

The scope for this work widens to the biotechnological sector where MUP may be designed to bind a variety of non-natural ligands that can be used as recognition sites for biosensors or gas sensors^10^. Because of their conformational stability, as well as resistance to degradation in the environment, this is opening a new pathway to designing biohybrid sensors capable of detecting a variety of analytes^27^. It is shown here that a single substitution of an amino acid involved in the binding process can radically change the MUP binding ability and ligand specificity. Our results pave the way to express different MUPs significantly different in sensitivity and selectivity for binding to a variety of volatile ligands, so that large arrays of proteins can be constructed, immobilised on to appropriate transducers to produce artificial sensing systems that are robust and suitable for applications such as environmental monitoring, medical diagnostics and food quality evaluation. Other researchers are actively pursuing the expression of olfactory receptor proteins and interfacing of nanoelectronic devices to produce promising chemical sensors^59–63^. However, because these proteins are membrane bound proteins, it has been difficult to retain the conformational stability needed to create robust sensors. The use of soluble and stable biorecognition elements such as MUPs provides a new tool for development of new chemical sensors.

## Methods

### Protein-Ligand Interactions

The NMR solution structure of MUP20 isoform^25^ (PDB ID: 2L9C) in the ligand-free state was used as the starting point for preliminary ligand docking simulations, which were run using the LPC/CSU Server – Weizmann AC and SwissDock (http://www.swissdock.ch). Prior to docking, the key residues putatively important for ligand binding were identified starting from the three-dimensional structure of the complex of MUP4 with 2-sec-butyl-4,5-dihydrothiazole (PDB ID: 3KFF)^35^. MUP4 shares high sequence homology (91% homology, 81% identity) and structural similarity with MUP20, especially in the ligand-binding pocket, where 16 out of 18 amino acids are conserved in the two proteins (the two different amino acids being L73 → S in MUP4 and E122 → F in MUP4). This information was transferred to the MUP20 structure. The Ligand-Protein Contacts and Contacts of Structural Units (LPC/CSU)^64^ were obtained for each atom–atom contact and ranked selecting those residues represented by nearest distance (Å) between atoms of the ligand and the MUP amino acids and largest contact surface (Å^2^)^38^. Potentially destabilising contacts were also identified. Residues with significant contact were selected for mutation in order to modulate the affinity of MUP for selected ligands. Values of Molecular Volume and LogP were determined using the online Molinspiration software version 2011.06 (www.molinspiration.com) and ALGOPS2.1 software.

### Selection of single point mutations in MUP20

A total of six amino acids (V59, L88, Y103, N107, L124, E137) with strong atomic interactions within protein-ligand complex were selected. Of these, 3 are conserved in all natural MUPs (Y103, N107, L124) and 3 are not (V59, L88, E137). They were mutated computationally to generate seven mutants (V59T, L88Q, Y103D, Y103R, N107L, L124V, E137K). The molecular modelling framework software Sirius 1.2 (San Diego Supercomputer Center, USDC) was used to visualize the 2L9C.pdb NMR solution structure of the wild type MUP20, in which the selected mutation sites were identified. After the single amino acid mutation in the structure of the protein, the mutants were evaluated for conformational stability using PyMOL software^43,65^. Structural comparison with original MUP20 3D model, including sequence alignment, structure alignment and RMSD statistics was carried out using SuperPose Version 1.0 (see Supplementary material S1)^66^.

#### Ligand selection and *in silico* docking

Protein and ligands were prepared and then blind docking experiments were performed using Autodock Vina (Molecular Graphics Laboratory, Department of Integrative Structural and Computational Biology, The Scripps Research Institute, La Jolla, CA, USA)^42,43^.

Output data (energies, interaction patterns) were analyzed and scored using UCSF Chimera molecular viewer^67^ and BIOVIA Discovery Studio software (Dassault Systèmes BIOVIA, San Diego), which were also used to produce the artworks^68^.

The ZINC database^69^ provided molecules in 3D format for ready-to-dock virtual screening.

#### Expression and Characterisation of mutant proteins

Selected mutant proteins were expressed *in vitro*. Salts, solvents and reagents were of analytical grade and purchased from Sigma (St. Louis, MO, USA), unless otherwise specified. Isopropyl-β-D-thiogalactoside and ampicillin LB-Media were from BioChemica, EuroClone, Milan, Italy.

Protein Expression: The wild type 6xHis-tagged MUP20 was designed with the addition of a 6xHisTag N-terminal tail in a pEAX plasmid (see Supplementary Material S2a), the resulting gene 6His-MUP20 was synthesised (Eurofins, Milan, Italy). The plasmid carrying the MUP20 was NdeI/EcorI digested and the insert genes were then recombined in pET5b vector for heterologous expression in DE3-BL21 E. coli (Supplementary Material S2b). After induction of bacterial expression with 0.4 mM IPTG, the expressed protein was confirmed by SDS-page based on the molecular weight. The same procedure was used for the expression of the mutants MUP20V59T, MUP20L88Q, MUP20L124V, MUP20Y103R, MUP20Y103D, MUP20N107L and MUP20E137K from genes obtained by site specific mutagenesis, validated by Sanger sequencing, in which the signal peptide (19 amino acids: MKLLVLLLCLGLTLVCVHA) was deleted from the sequences.

Protein purification: The His tag system (HisTrap™ FF crude, GE Healthcare Bio-Sciences) was used to purify the mutant proteins. A HisTrap column was eluted in 20 mM sodium phosphate buffer, pH 7.4, containing 0.5 M NaCl and 0.5 M imidazole. The eluted protein was delipidated on a Sephadex LH-20 column, eluted with 50 mM acetate buffer (pH 4.5). Delipididated protein were dialyzed overnight against 50 mM Tris-HCl buffer, pH 7.4, containing 0.15 M NaCl, and stored at −80 °C.

Chemical characterization of the mutant MUPs: The homogeneity of MUP mutants was analysed by SDS-PAGE (12% acrylamide), under reducing conditions, after Coomassie staining. RP-HPLC analyses were run on a Grace-Vydac (Hesperia, CA, USA) C4 analytical column (4.6 × 150 mm). The column was eluted with a linear 0.1% (v/v) TFA-acetonitrile gradient from 10 to 60% in 30 minutes at a flow rate of 0.8 ml/min and the absorbance of the effluent was recorded at 226 nm. The chemical identity of the purified mutant MUPs was established by high-resolution mass spectrometry on a Xevo G2-S Q-TOF mass spectrometer (Waters, MA, USA). The proteins were also analysed for possible volatile contaminants via Solid Phase Micro Extraction (SPME) of the headspace followed by GC/MS analysis.

Spectroscopic measurements: The protein concentrations were determined spectrophotometrically, by measuring the absorbance at 280 nm, on a V-630 spectrophotometer (Jasco, Tokyo, Japan) using an absorptivity value of 1.618 mg^−1^∙cm^2^. Circular dichroism (CD) spectra were recorded on a Jasco J-810 spectropolarimeter equipped with a thermostated cell holder and a Peltier PTC-423S temperature control system. Spectra were recorded using 0.1- or 1-cm pathlength cuvettes in the far- and near-UV region, respectively. Each spectrum was the average of four accumulations, after baseline subtraction. Ellipticity data were expressed as the mean residue ellipticity, [Θ] = (Θ∙MRW)/(10∙l∙c), where Θ is the measured ellipticity in degrees, MRW is the mean residue weight, l is the cuvette pathlength, and c is the protein concentration in g/ml^70^. All measurements were conducted in PBS buffer (10 mM Na_2_HPO_4_ pH 7.4, 0.15 M NaCl) at 25 ± 0.1 °C.

Fluorescence competitive binding assays: Fluorescence-competitive binding assays were carried out using the fluorescent probe N-phenyl-1-naphthylamine (1-NPN), as described^39,40^. A binding curve was constructed and the dissociation constant of the probe (K_D_) was calculated. Proteins were diluted to 1 μM in 50 mM Tris-HCl, pH 7.4, then 1.6 μM of the fluorescent probe 1-NPN was added and equilibrated for 1 minute. The solution was excited at 337 nm and the emission spectra were measured at 25 °C in the wavelength range 380–450 nm on a Perkin Elmer LS50 pulsed lamp spectrofluorimeter.

The binding analyses assumed that the protein had 100% activity and that the stoichiometry of binding was 1:1 at saturation. The affinity of various volatile ligands was measured in competitive binding assays.

The change in fluorescence was recorded after adding increasing concentrations of ligand. Competitor concentrations causing a decay of fluorescence to half-maximal intensity were determined as IC50 and the dissociation constant for the test ligands (expressed as K_d_) was calculated using the following formula:

1 $${{\rm{K}}}_{{\rm{d}}}=\frac{[{\rm{IC50}}]}{1+\frac{[{\rm{Probe}}]}{{{\rm{K}}}_{{\rm{D}}}}}$$

where [Probe] is the free concentration of the fluorescent probe and K_D_ is the dissociation constant of the complex Protein/Probe.

Isothermal Titration Calorimetry (ITC): The binding energetics of the best binding ligands, 2-isobutyl-3-methoxypyrazine (PYR) and menadione (MEN), to wild-type (WT) and mutant MUPs were studied. ITC titrations were performed at 25 ± 0.1 °C in 50 mM Tris HCl pH 7.4, using a MicroCal VP-ITC instrument. To a MUP solution (1.7 ml, 25 µM) were sequentially added 25 aliquots (10 μl each) of a ligand stock solution (500 μM), with a delay of 4 min after each injection with continuous stirring of the solution (at 307 r.p.m.) in the sample cell. Before analysis, protein samples were dialyzed overnight in the same buffer, using a Slide-A-Lyzer (3.5-kDa cutoff) from ThermoFisher Scientific (Waltham, MA, USA), and thoroughly degassed. The heat of dilution was determined in control experiments by injecting aliquots (10 µl) of a ligand stock solution (500 µM) into buffer and this was subtracted from the integrated binding isotherm prior to curve fitting. Each injection generated a heat-burst curve (μcal s^−1^) versus time (min). The area under each peak was determined by integration using Origin 7.5 software (Microcal, Inc.) to give the heat associated with the injection. The heat exchanged per mole of injectant was plotted against the ligand:protein molar ratio. The binding constants (K_a_) and stoichiometry (N) parameters of the binding process were obtained by fitting the integrated heats of binding to the one-site binding model using the MicroCal ITC Data Analysis software.

## Electronic supplementary material

Supplementary Material S1 to S5.
