# Supplementary material for: Effects of point mutations in the binding pocket of the mouse major urinary protein MUP20 on ligand affinity and specificity
Source: Sci Rep. 2019 Jan 22;9:300. doi: 10.1038/s41598-018-36391-3 (PMC6342991; doi:10.1038/s41598-018-36391-3)
Supplement: Supplementary file 1 — Supplementary Material S1 to S5. [file 41598_2018_36391_MOESM1_ESM.pdf]

Effects of point mutations in the binding pocket of the mouse major urinary protein MUP20 on ligand affinity and specificity

J. Ricatti, L. Acquasaliente, G. Ribaudo, V. De Filippis, M. Bellini, R. Esteban Llovera, S. Barollo, R. Pezzani, G. Zagotto, K. C. Persaud, C. Mucignat-Caretta

Supplementary Material S1

Structural analysis and *in silico* comparison of MUP20 WT and mutants

Structural comparison, including sequence alignment, structure alignment and RMSD statistics was carried out using SuperPose Version 1.0 [Rajarshi Maiti. Gary H. Van Domselaar. Haiyan Zhang. and David S. Wishart "SuperPose: a simple server for sophisticated structural superposition" Nucleic Acids Res. 2004 July 1; 32 (Web Server issue): W590W594]. The resulting 3D superimpositions between MUP20 WT and the 7 mutants are reported in the stereographic figures below, together with RMSD data.

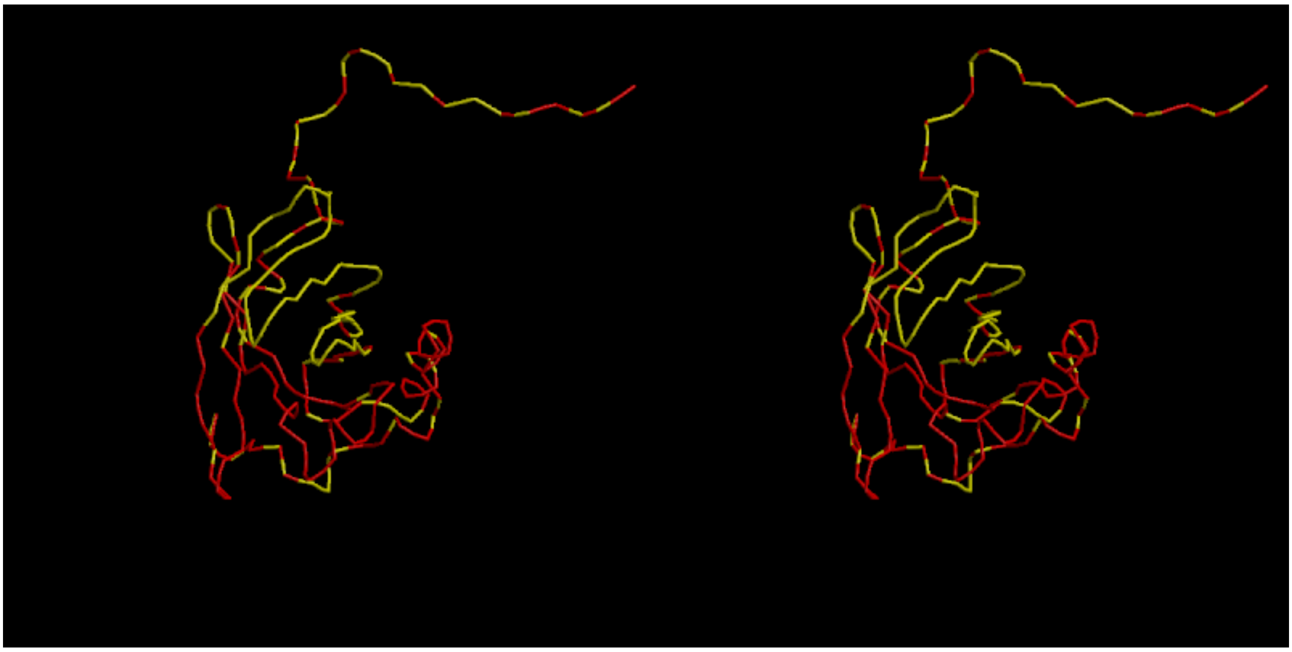

Stereographic 3D superimposition of MUP20 WT and V59T (RMSD = 0.05).

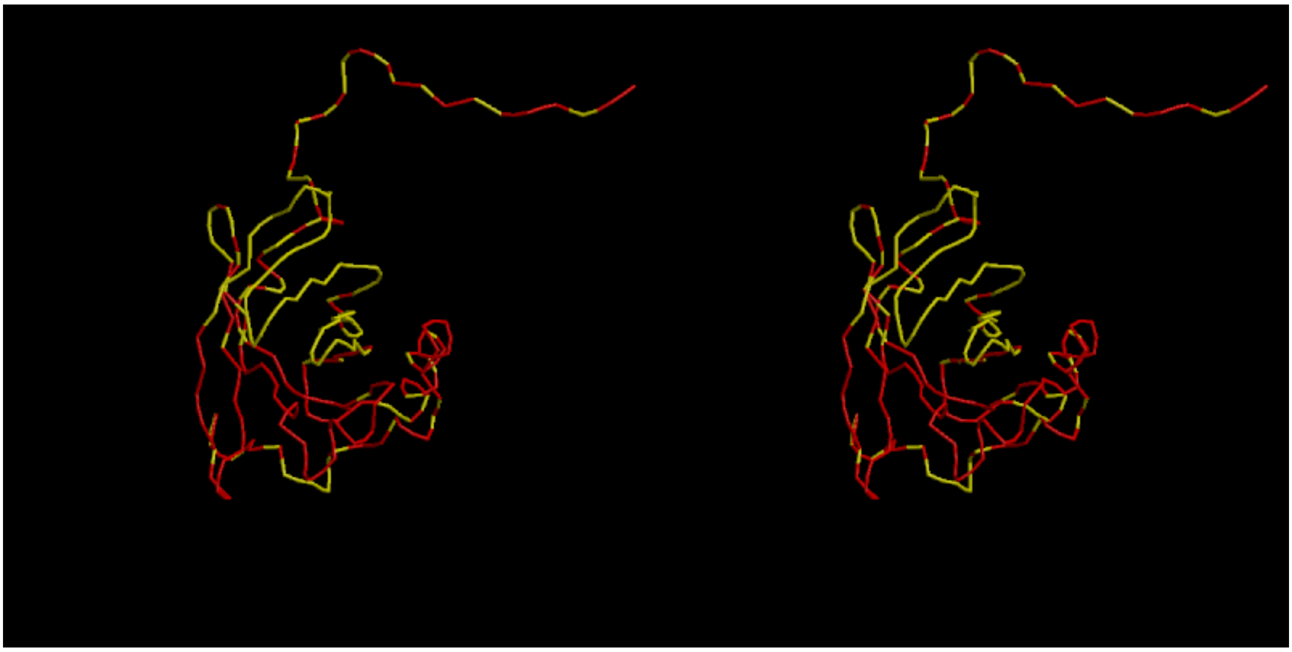

Stereographic 3D superimposition of MUP20 WT and L88Q (RMSD = 0.04).

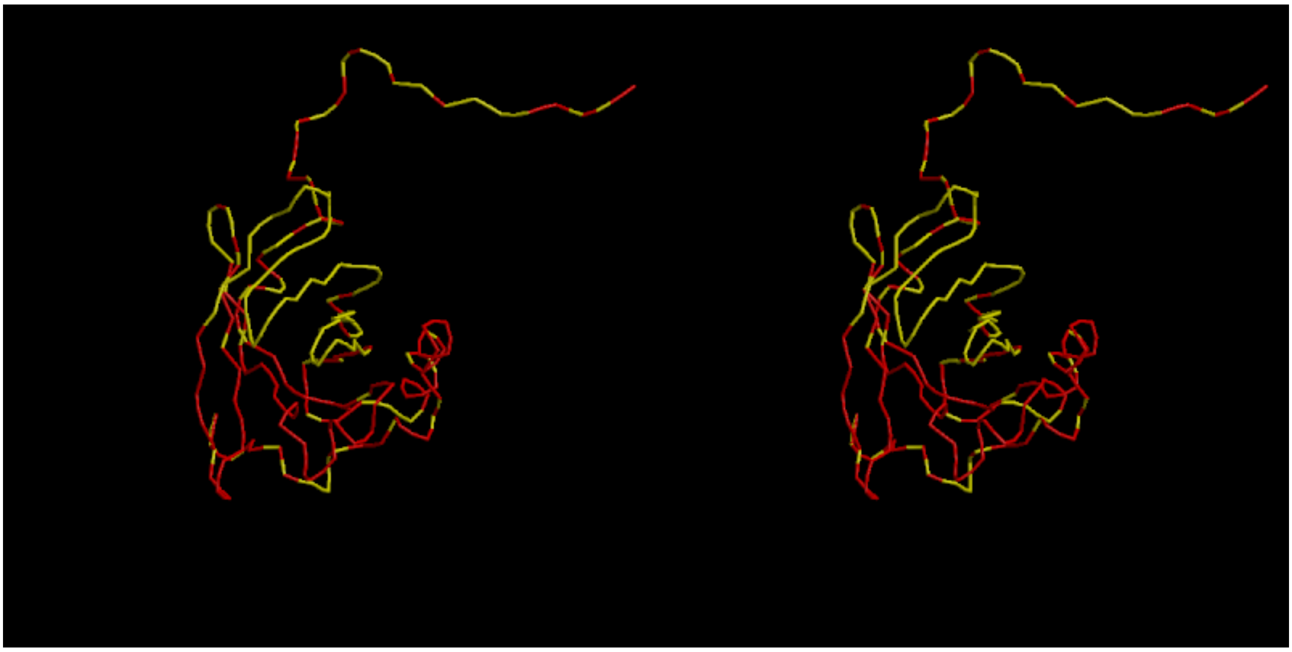

Stereographic 3D superimposition of MUP20 WT and Y103D (RMSD = 0.06).

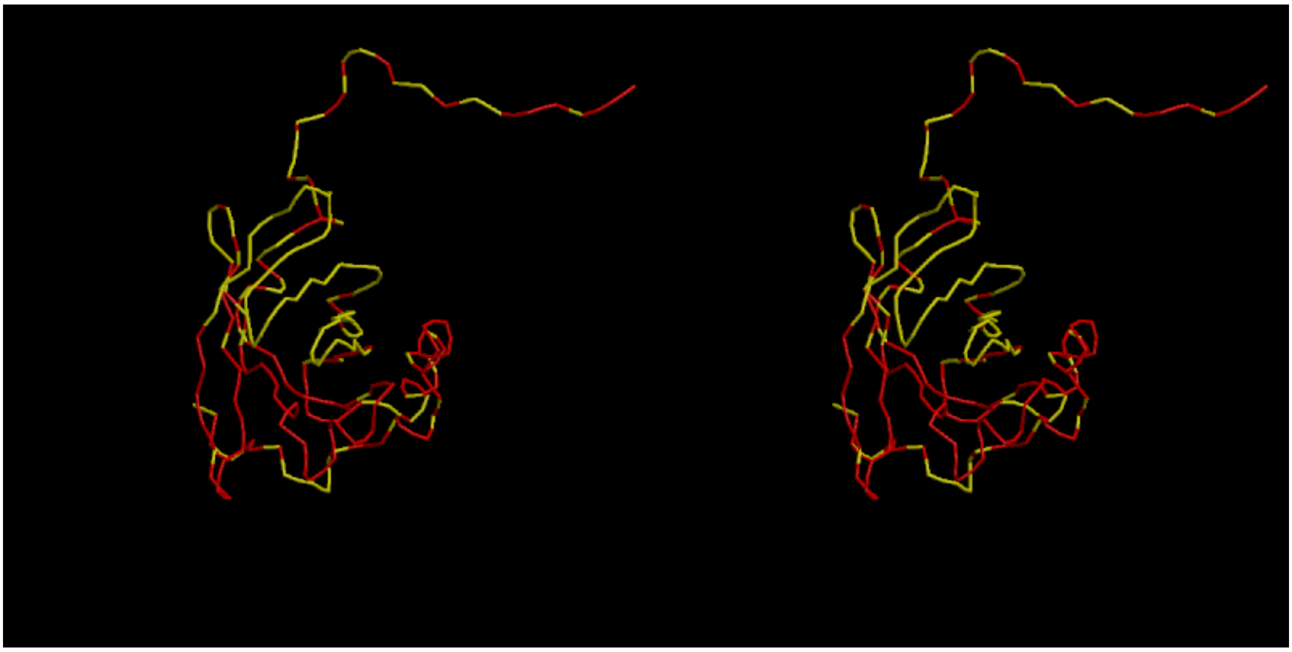

Stereographic 3D superimposition of MUP20 WT and Y103R (RMSD = 0.05).

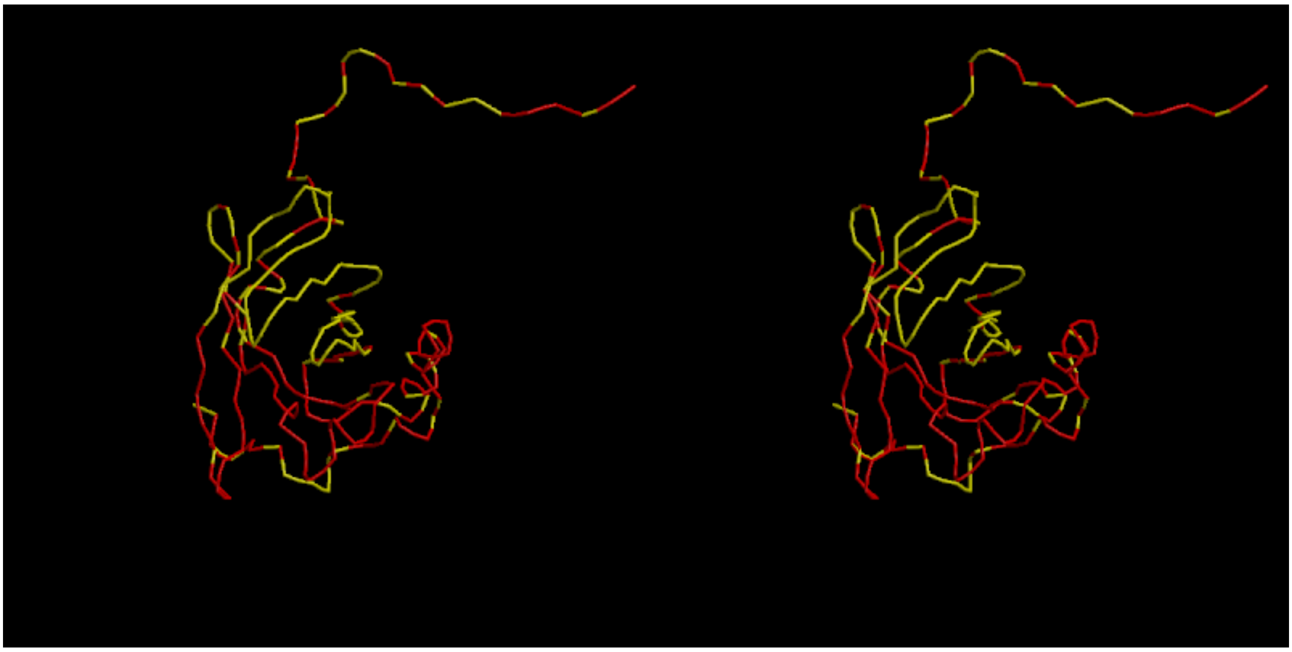

Stereographic 3D superimposition of MUP20 WT and N107L (RMSD = 0.01).

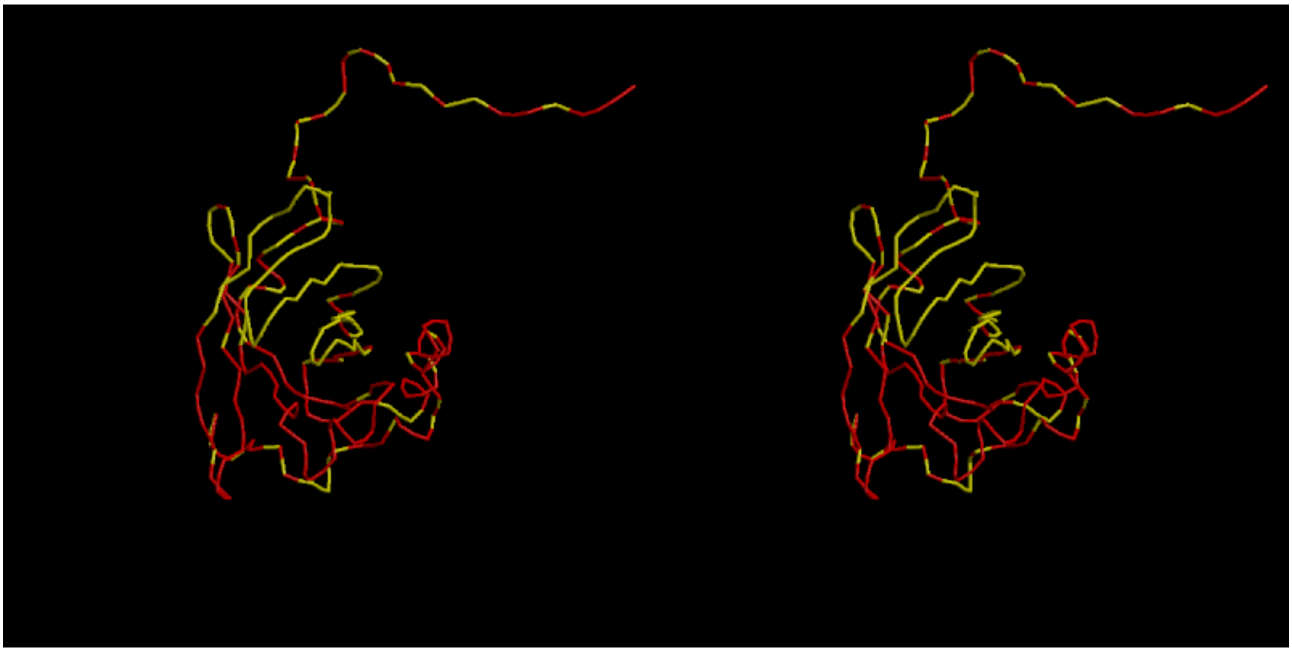

Stereographic 3D superimposition of MUP20 WT and L124V (RMSD = 0.01).

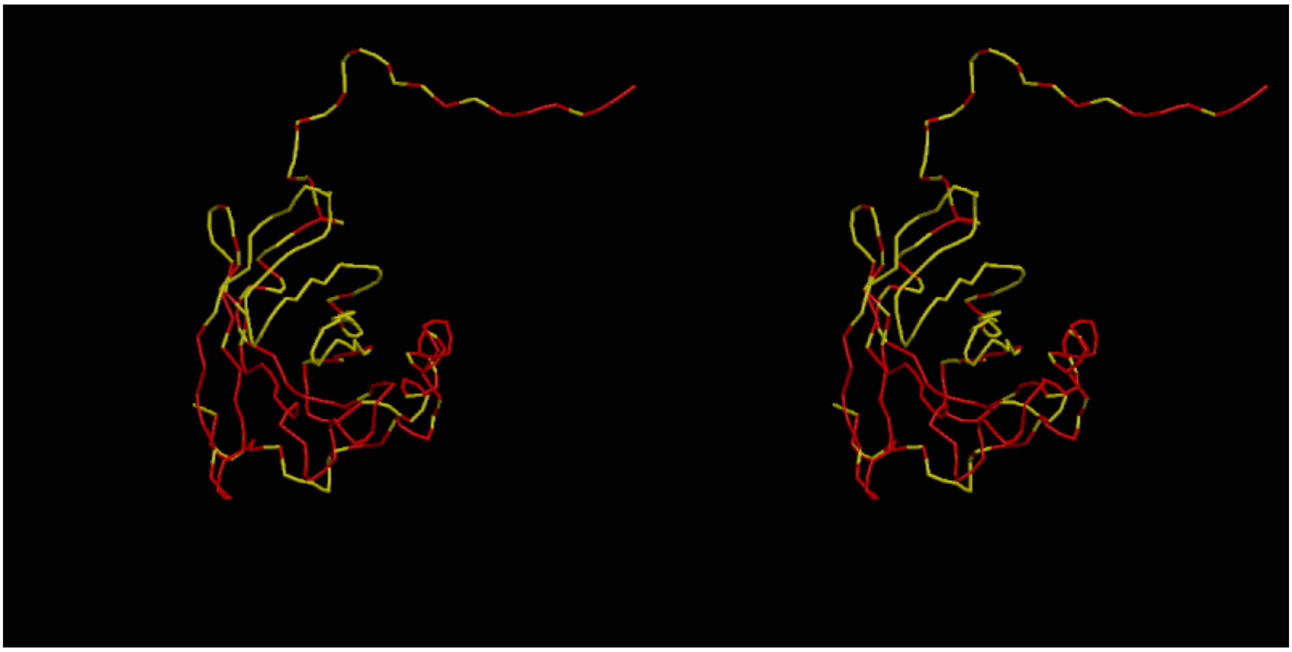

Stereographic 3D superimposition of MUP20 WT and E137K (RMSD = 0.09).

Calculated 2D interaction pattern of the ligands with MUP20 mutants

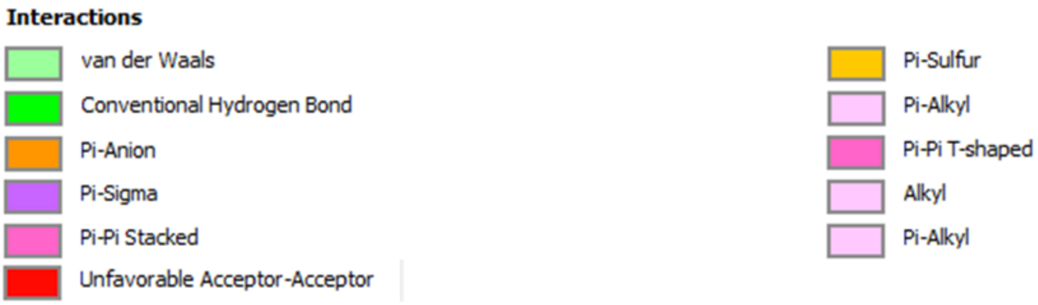

Color key of the represented interactions. Residue name and number of the interacting residues are shown in the pictures below, together with the distance between the ligand and the residue (expressed in Å). Pictures were obtained using Discovery Studio (Dassault Systèmes BIOVIA. San Diego).

MUP20 WT

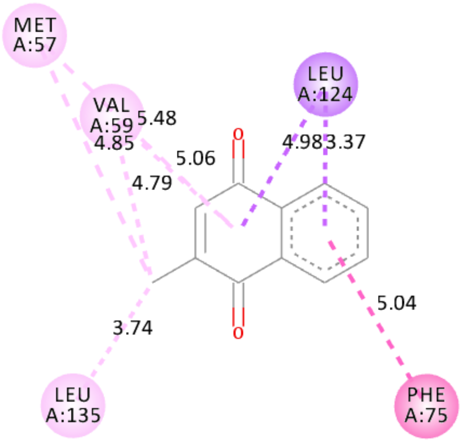

2D interaction pattern of MEN.

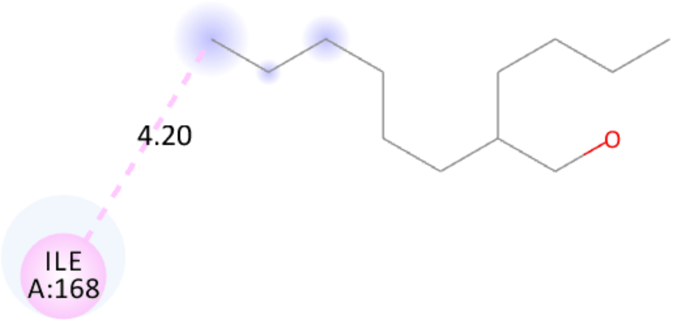

2D interaction pattern of OCT.

MUP20 WT

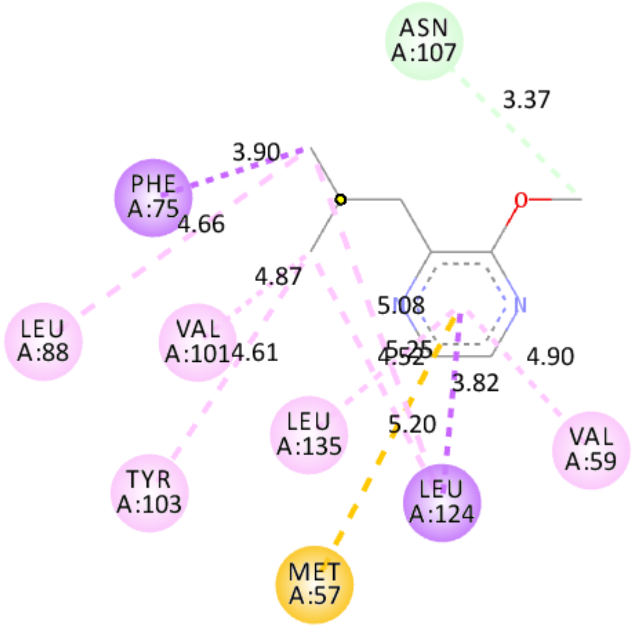

2D interaction pattern of PYR.

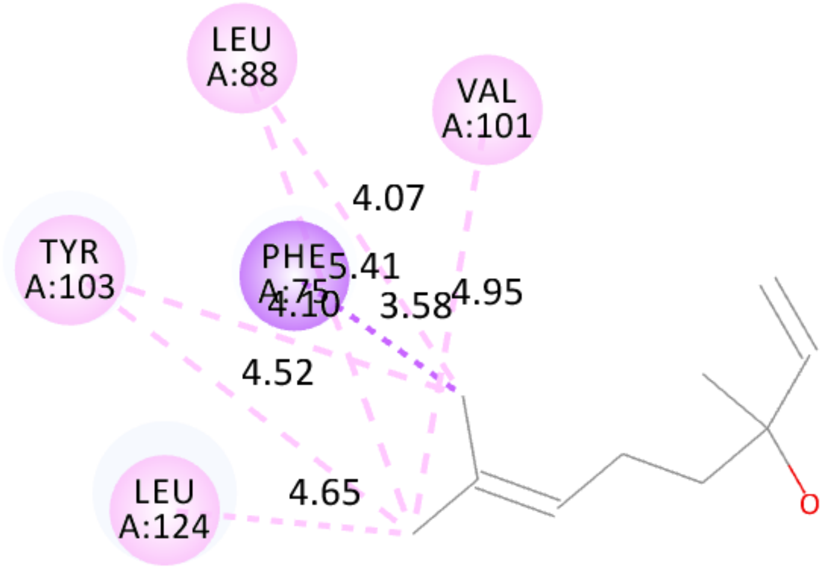

2D interaction pattern of LIN.

MUP20 WT

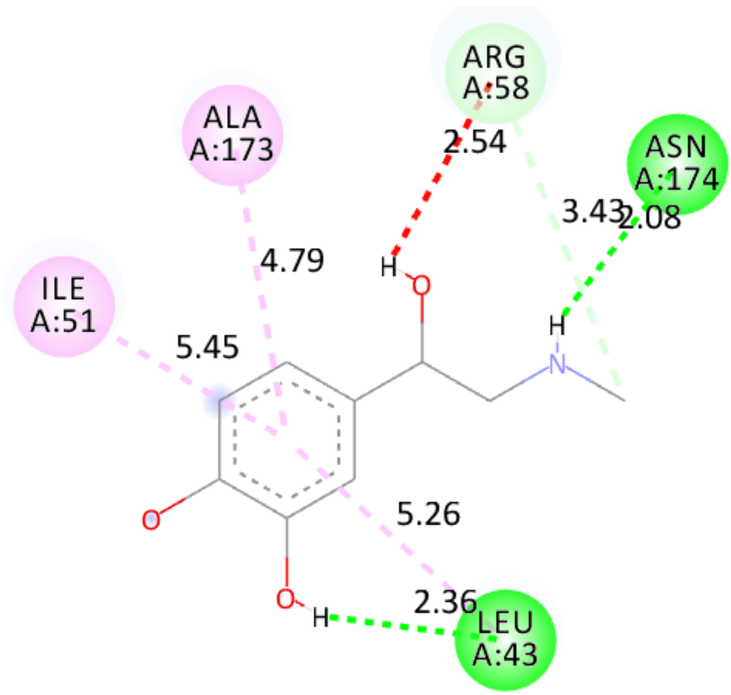

2D interaction pattern of L-ADR.

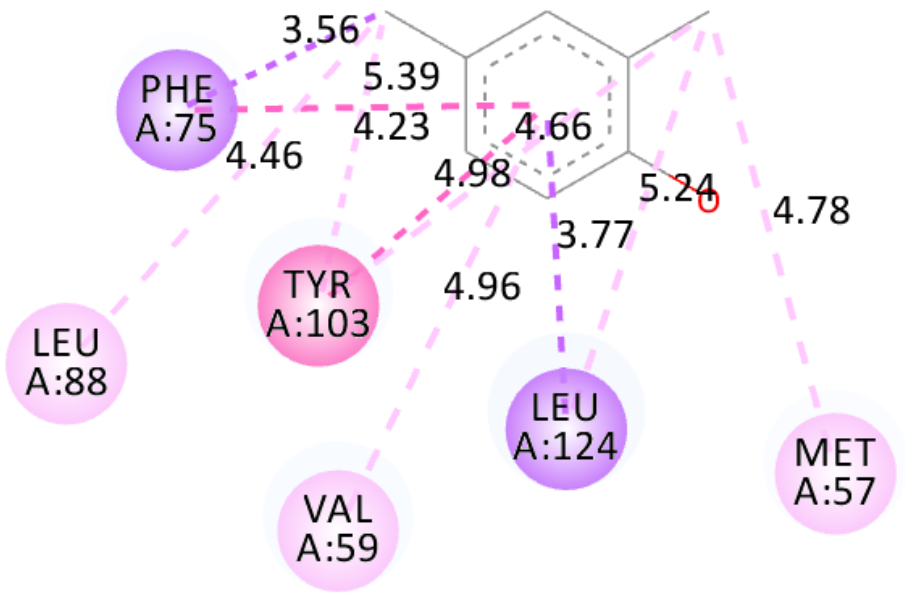

2D interaction pattern of 2,4-DMP.

V59T

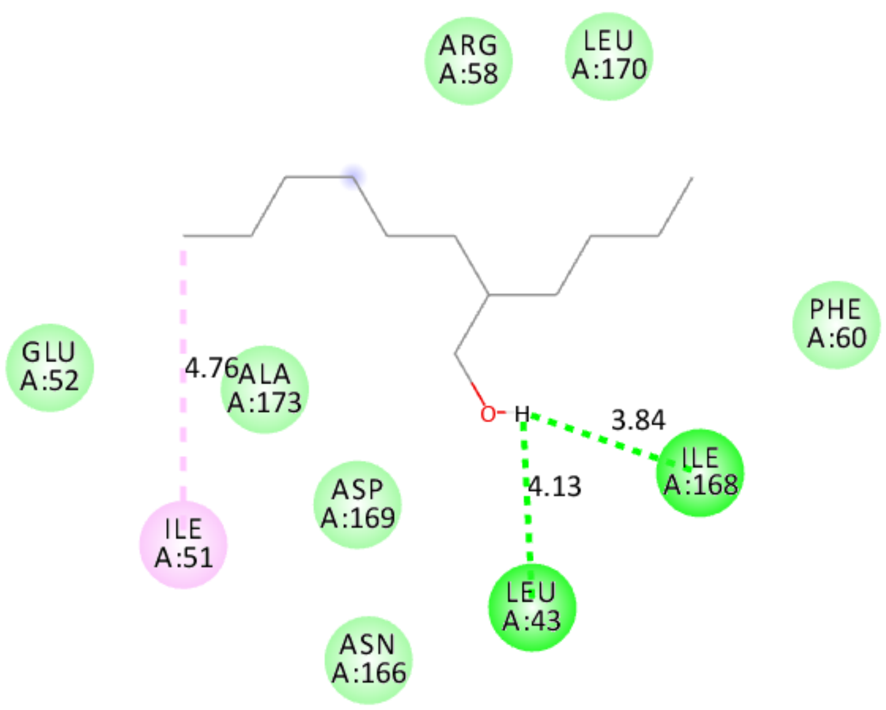

2D interaction pattern of OCT.

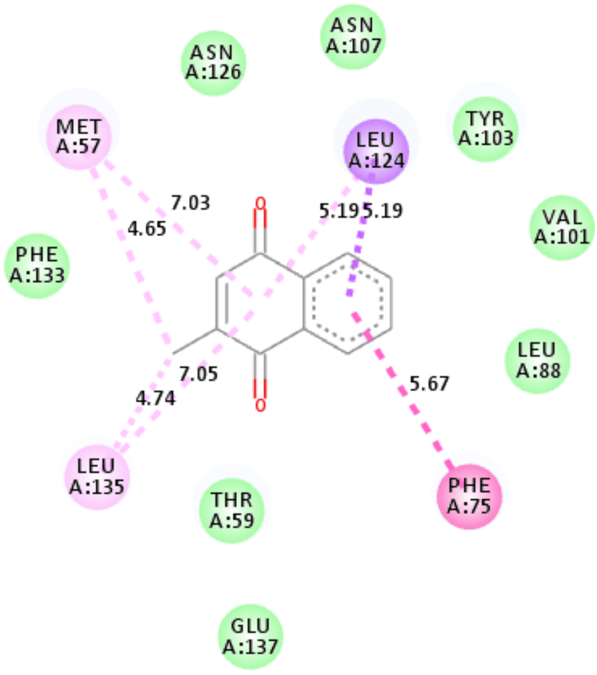

2D interaction pattern of MEN.

V59T

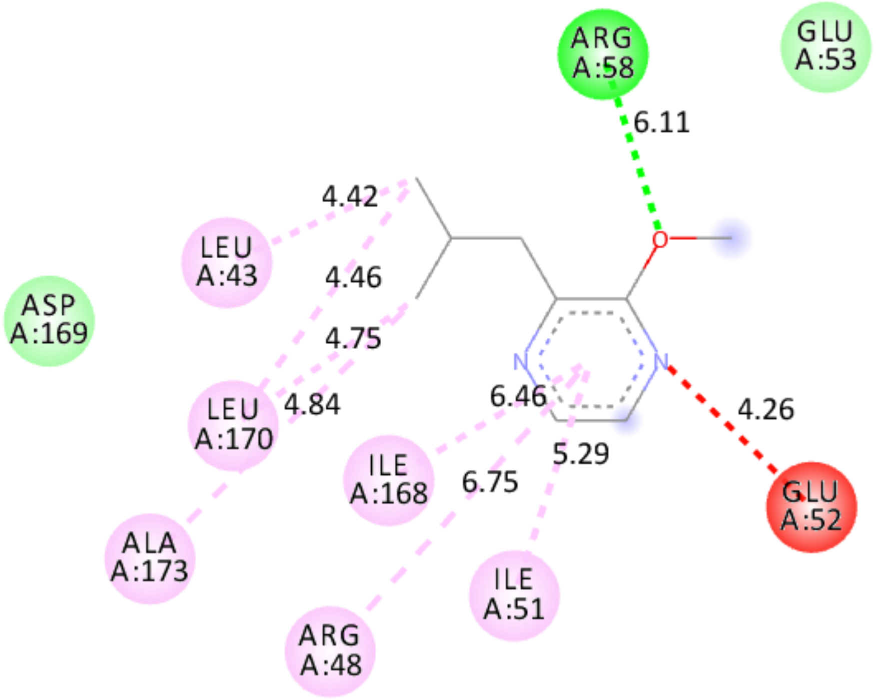

2D interaction pattern of PYR.

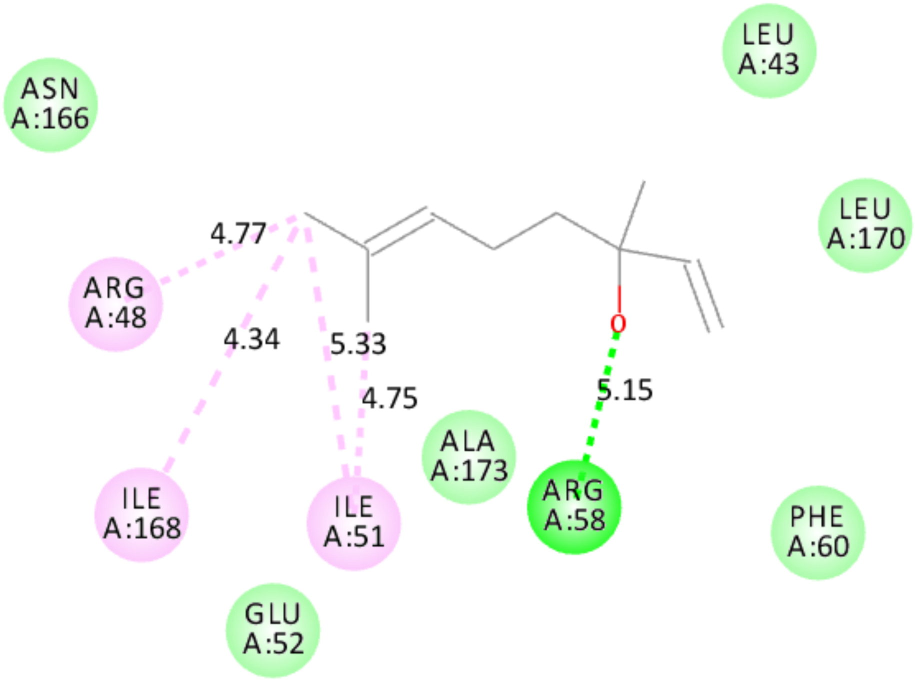

2D interaction pattern of LIN.

V59T

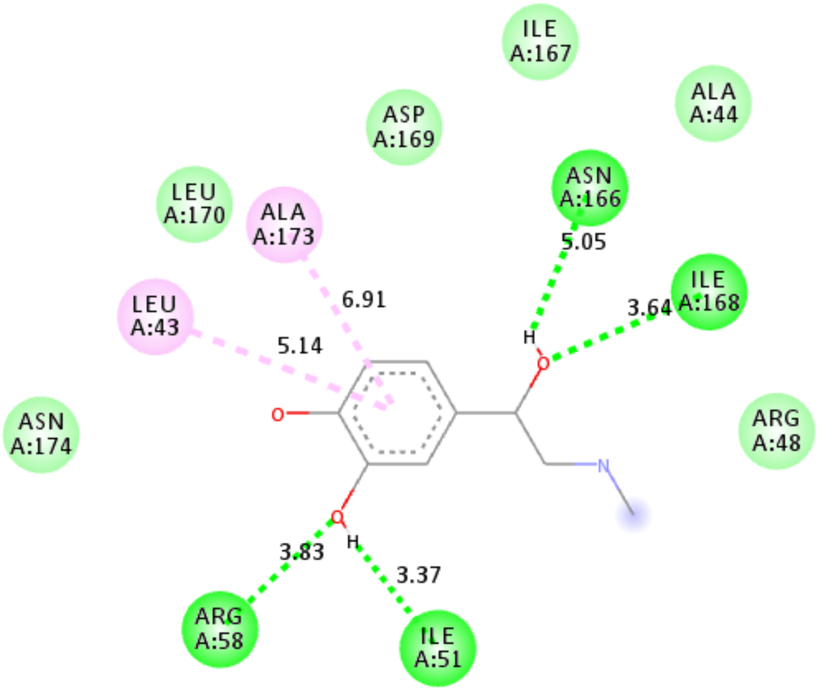

2D interaction pattern of L-ADR.

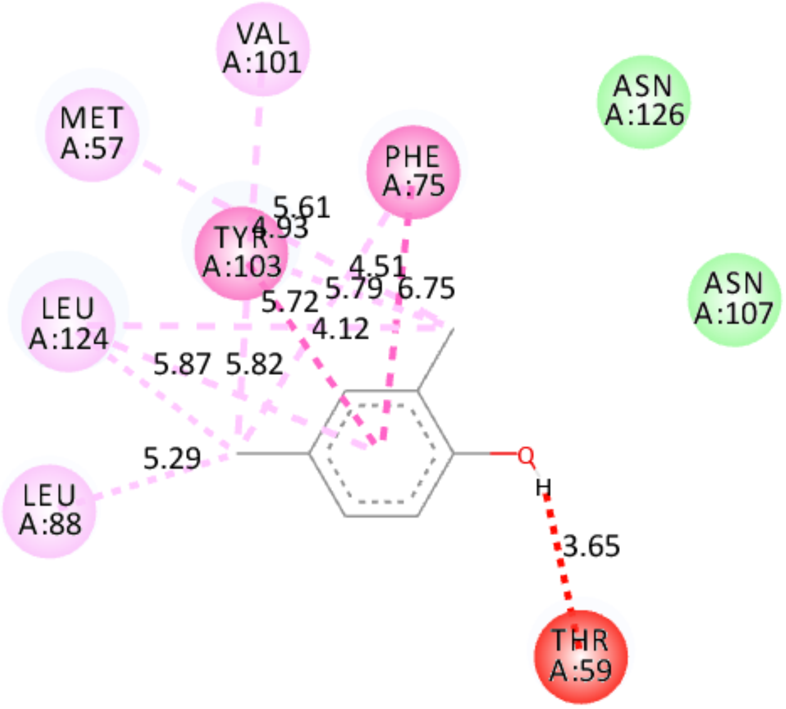

2D interaction pattern of 2,4-DMP.

L88Q

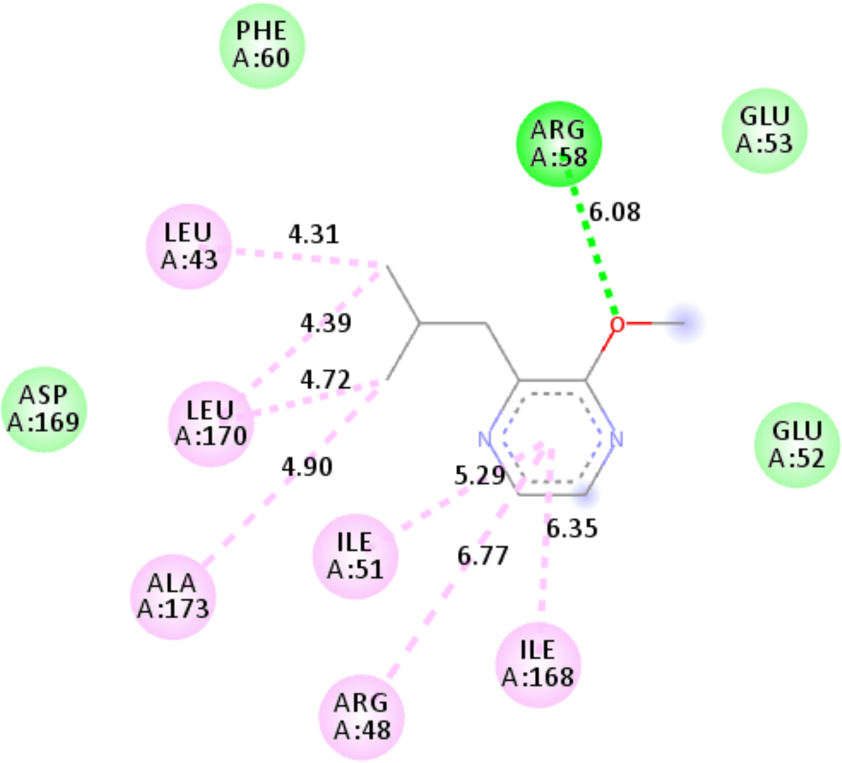

2D interaction pattern of PYR.

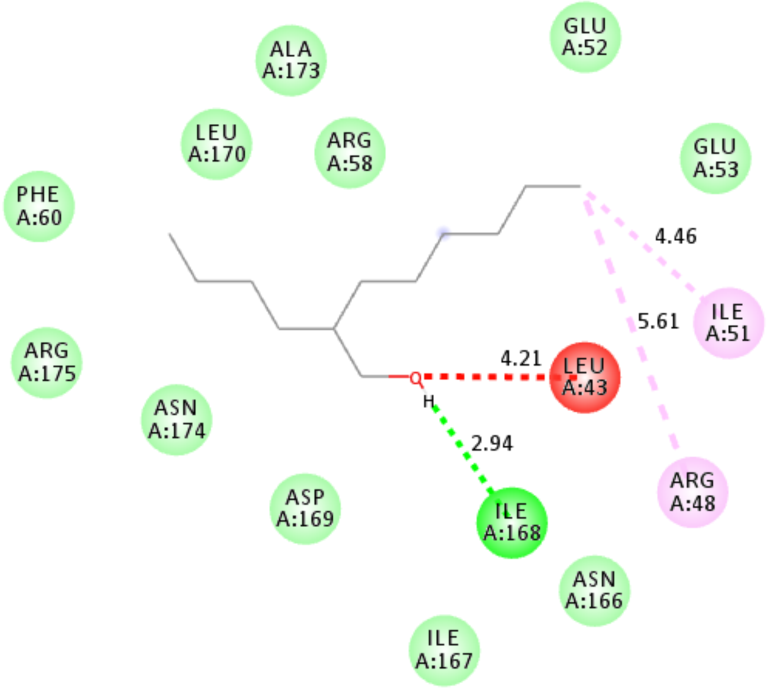

2D interaction pattern of OCT.

L88Q

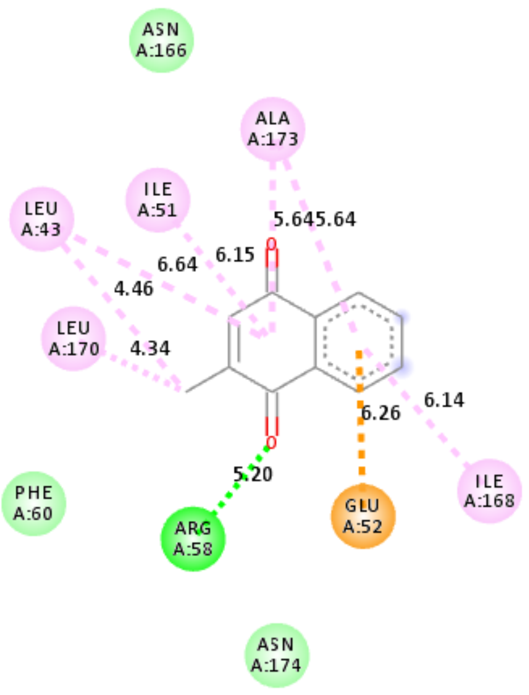

2D interaction pattern of MEN.

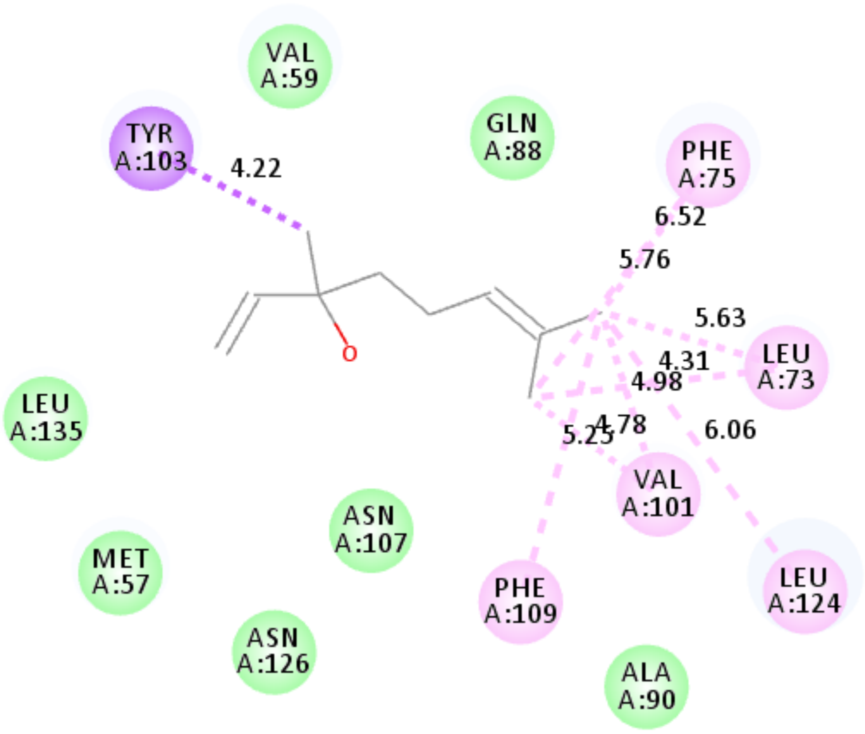

2D interaction pattern of LIN.

L88Q

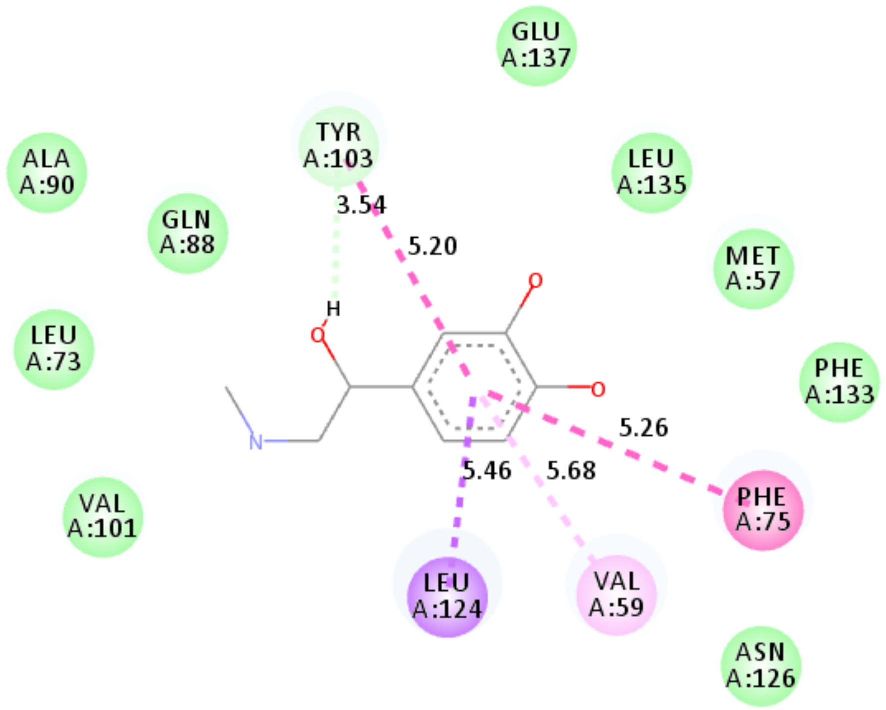

2D interaction pattern of L-ADR.

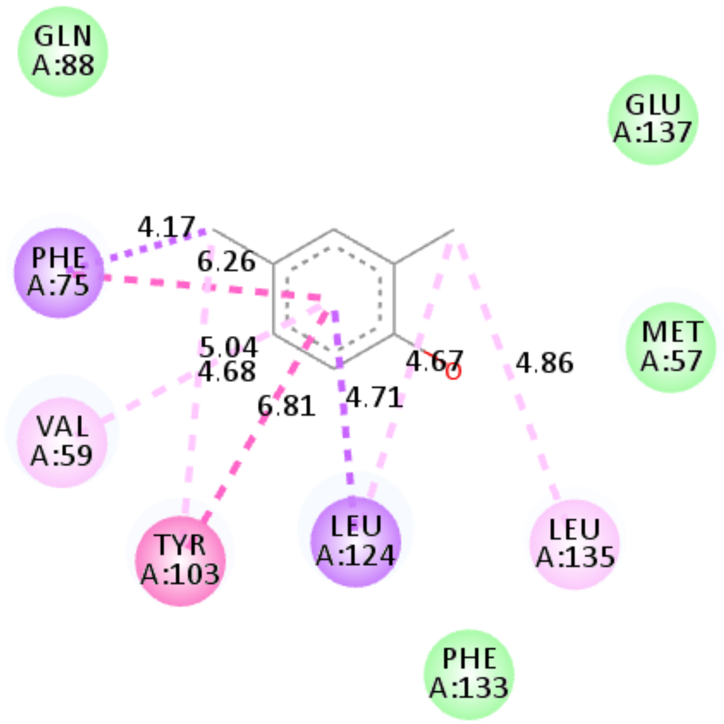

2D interaction pattern of 2,4-DMP.

Y103D

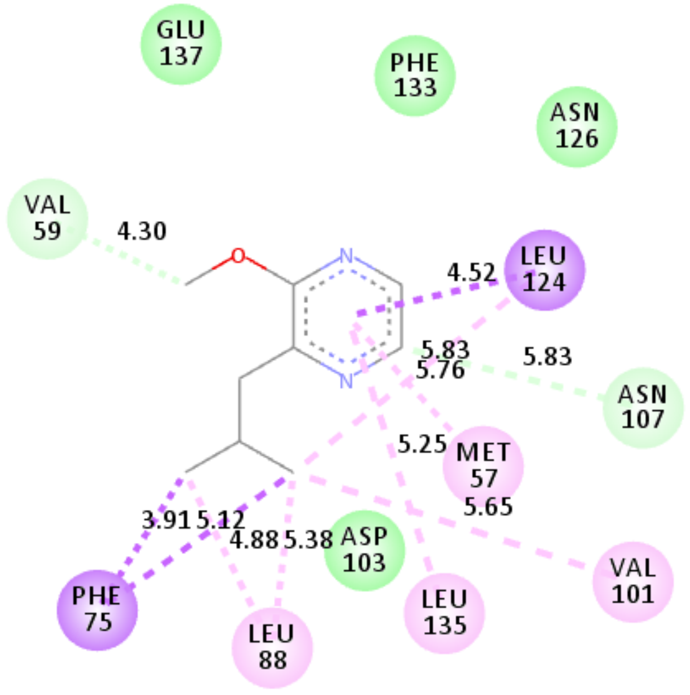

2D interaction pattern of PYR.

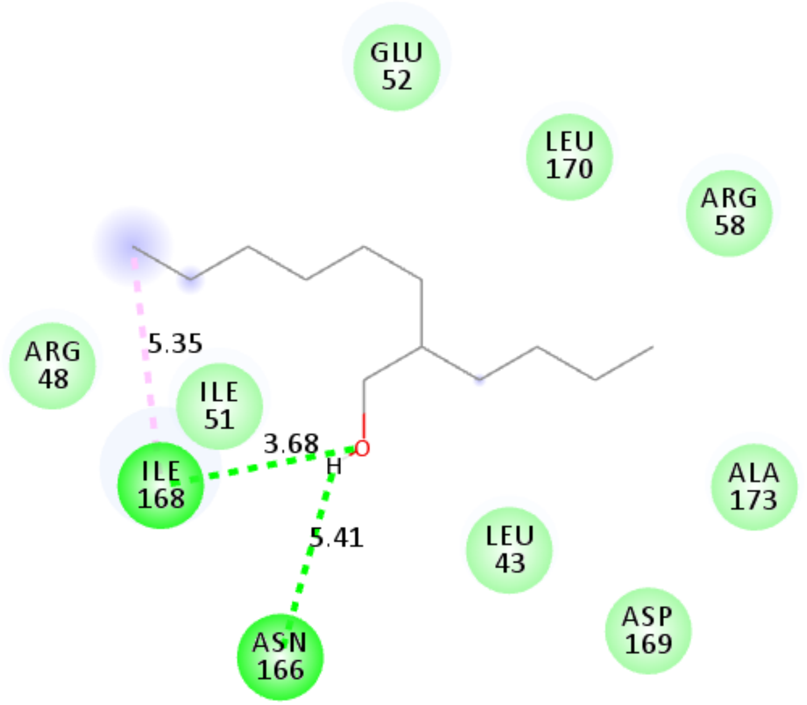

2D interaction pattern of OCT.

Y103D

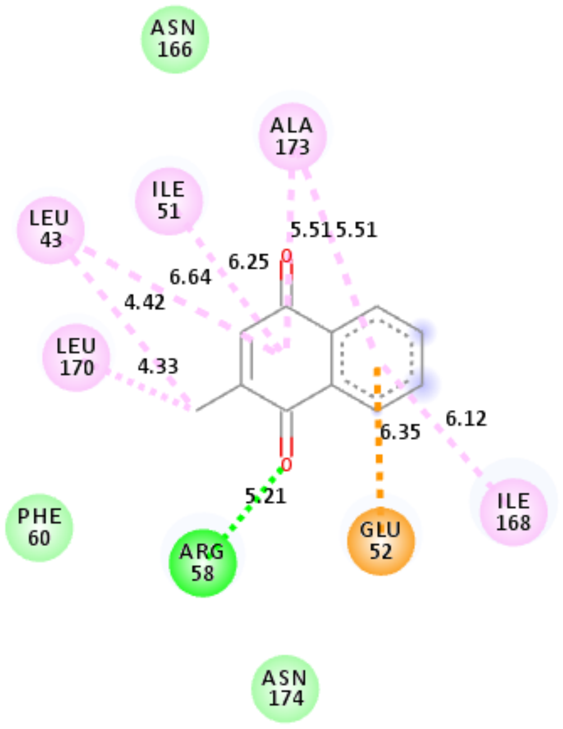

2D interaction pattern of MEN.

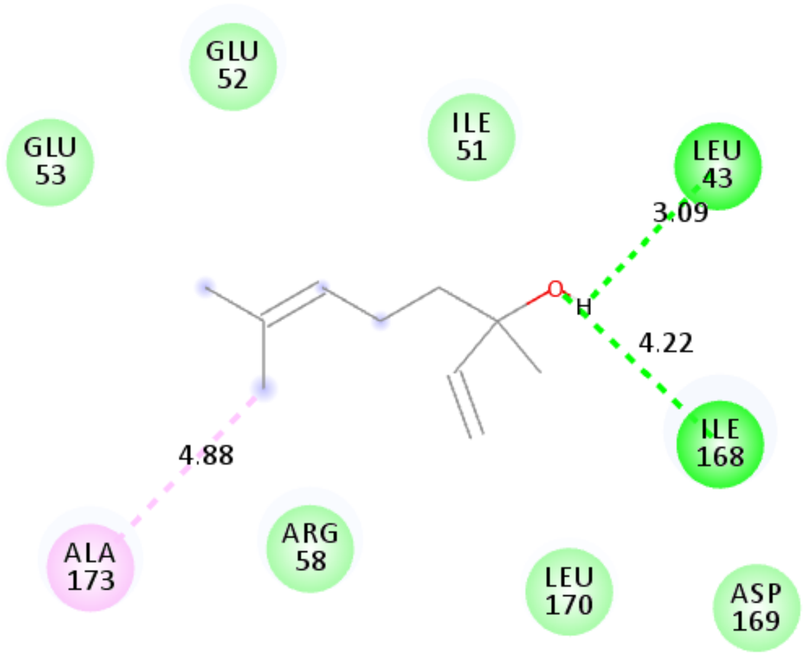

2D interaction pattern of LIN.

Y103D

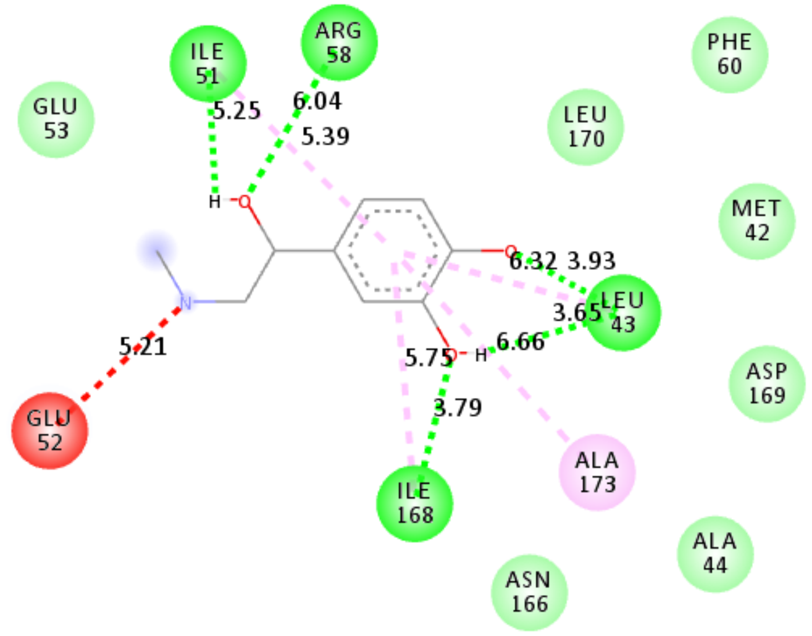

2D interaction pattern of L-ADR.

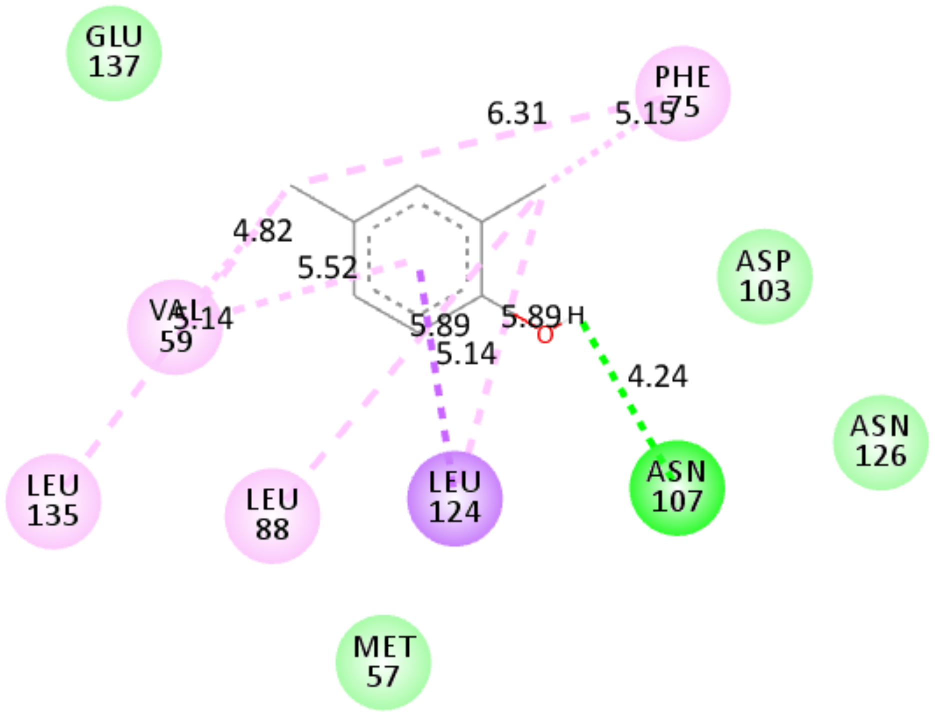

2D interaction pattern of 2,4-DMP.

Y103R

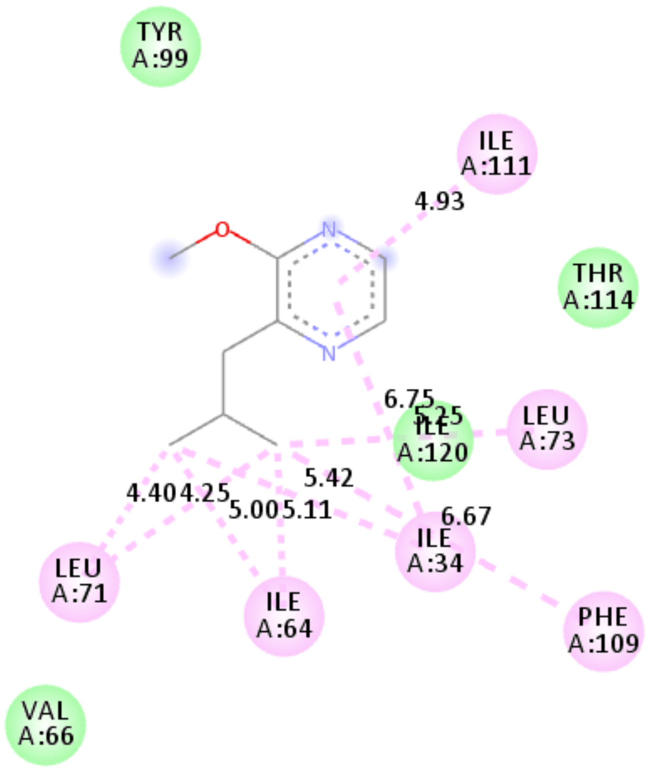

2D interaction pattern of PYR.

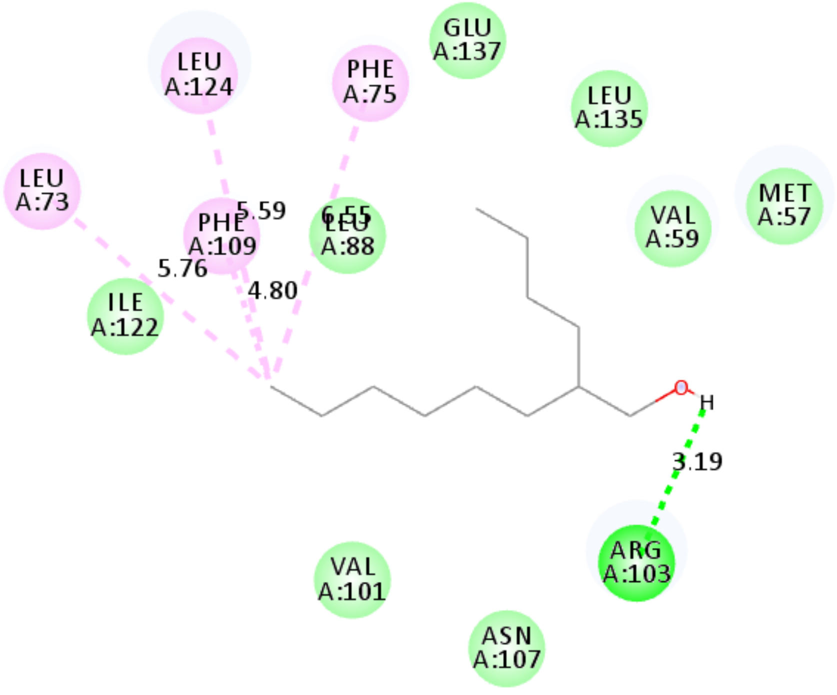

2D interaction pattern of OCT.

Y103R

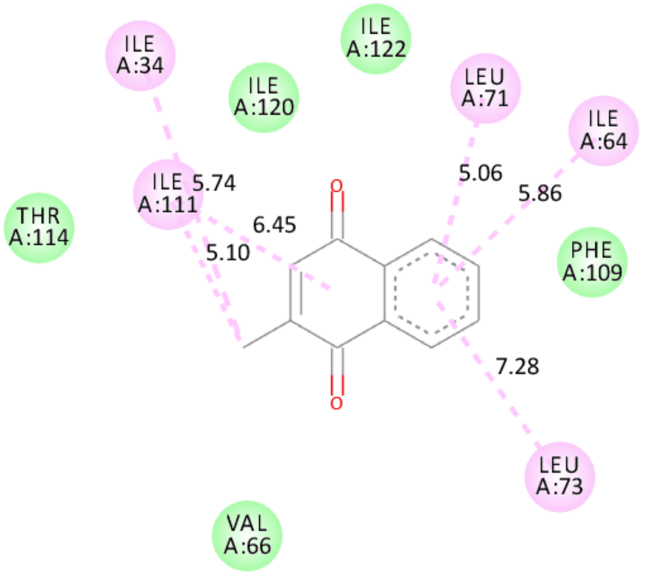

2D interaction pattern of MEN.

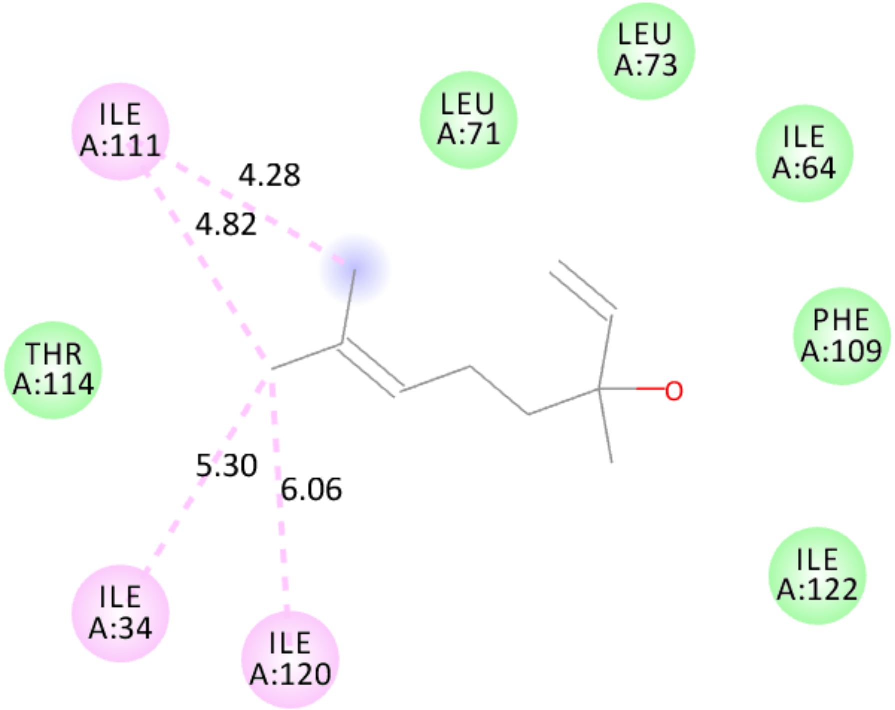

2D interaction pattern of LIN.

Y103R

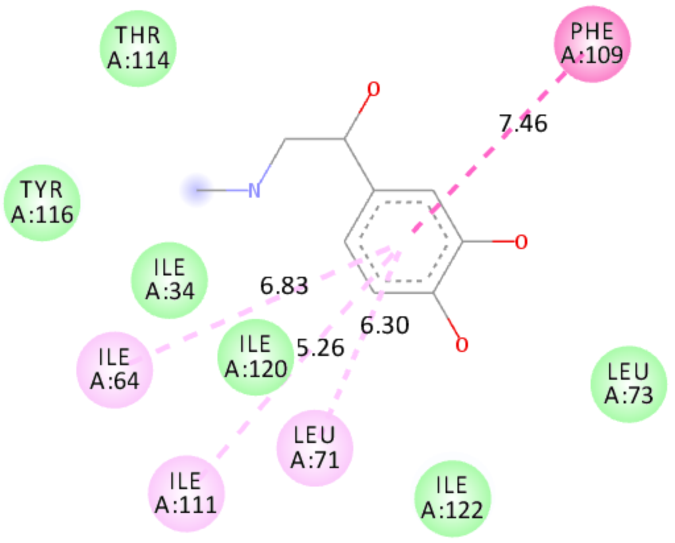

2D interaction pattern of L-ADR.

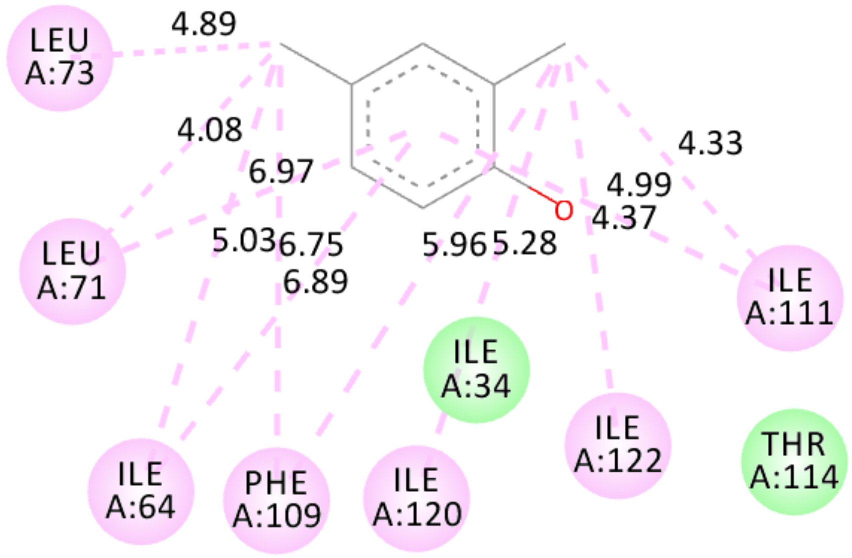

2D interaction pattern of 2,4-DMP.

N107L

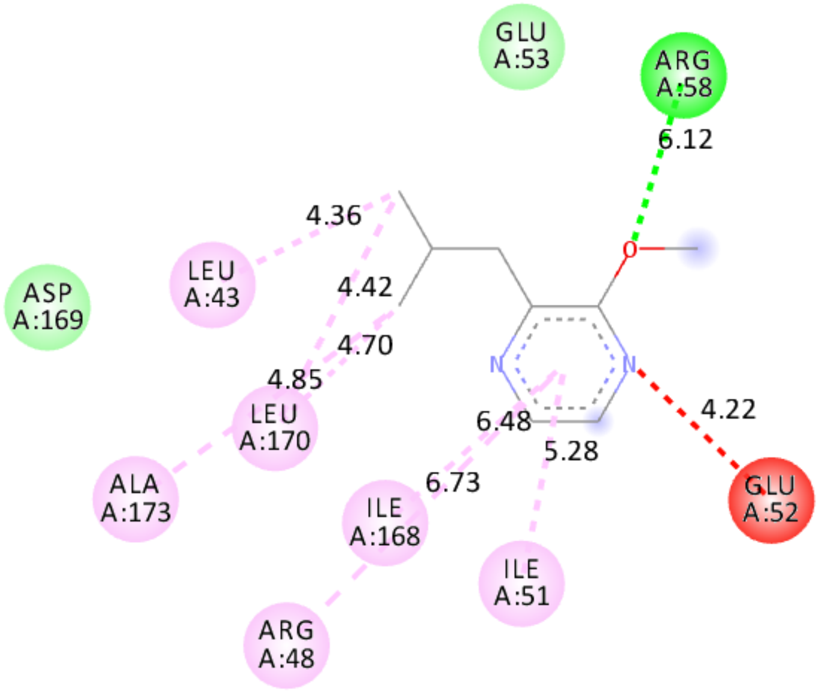

2D interaction pattern of PYR.

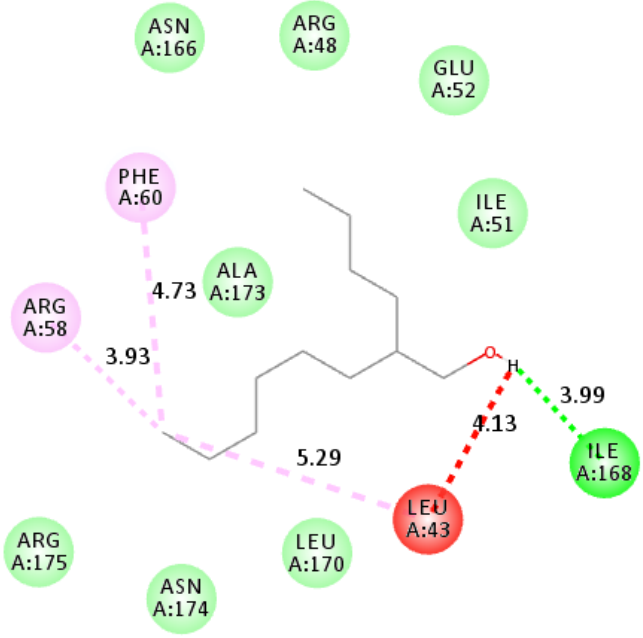

2D interaction pattern of OCT.

N107L

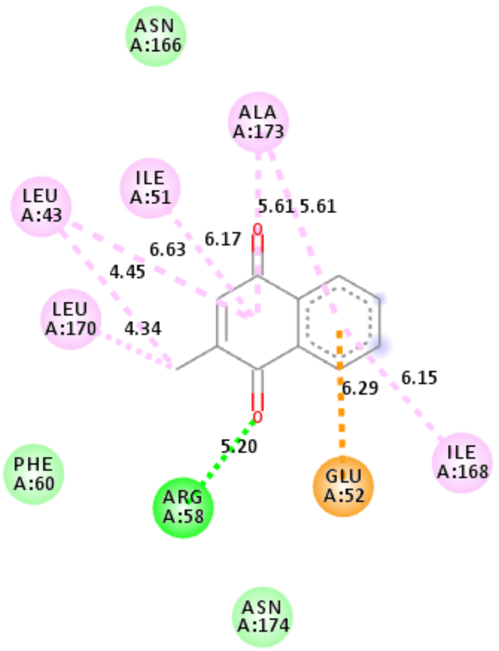

2D interaction pattern of MEN.

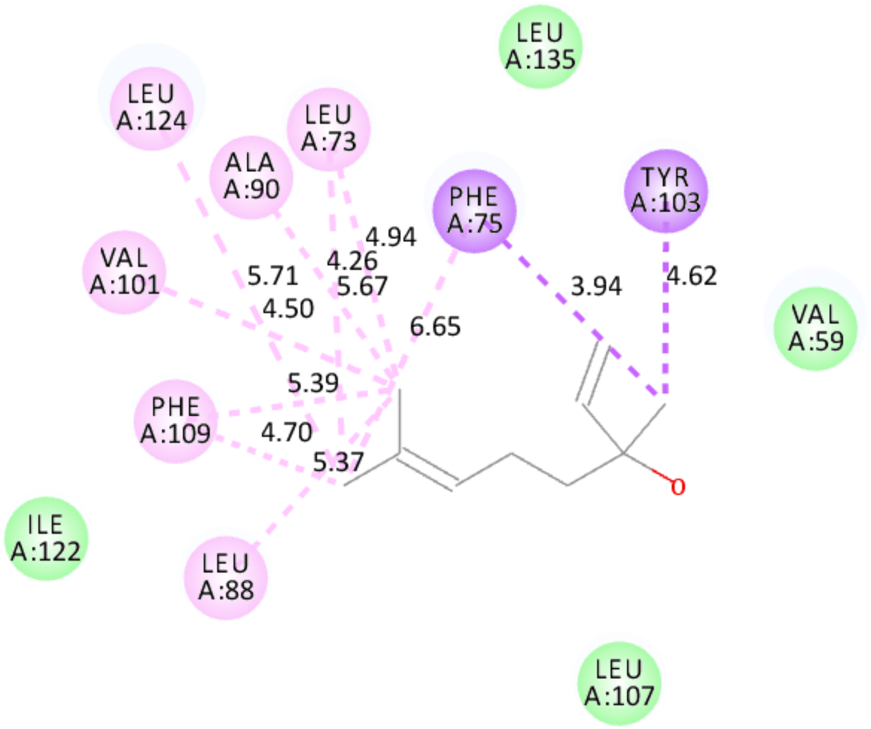

2D interaction pattern of LIN.

N107L

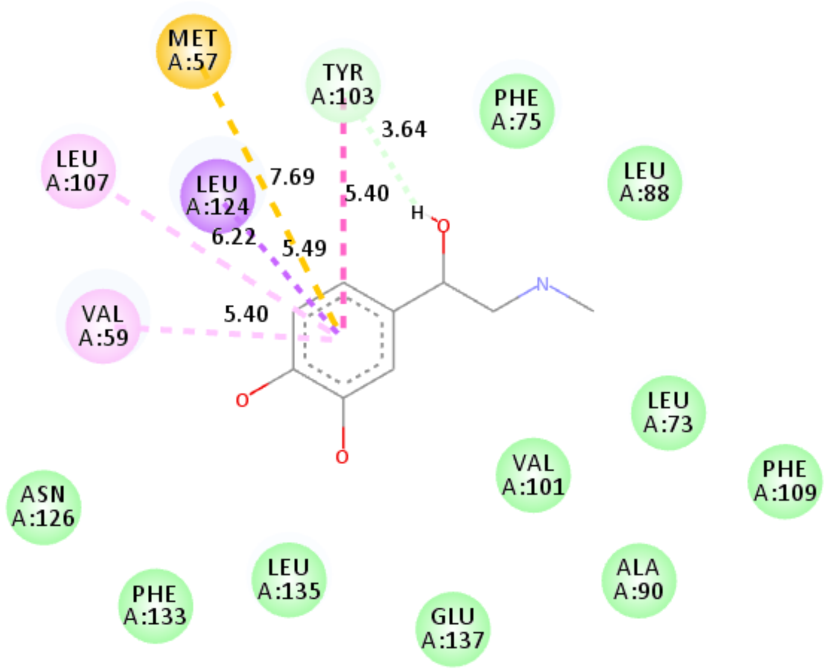

2D interaction pattern of L-ADR.

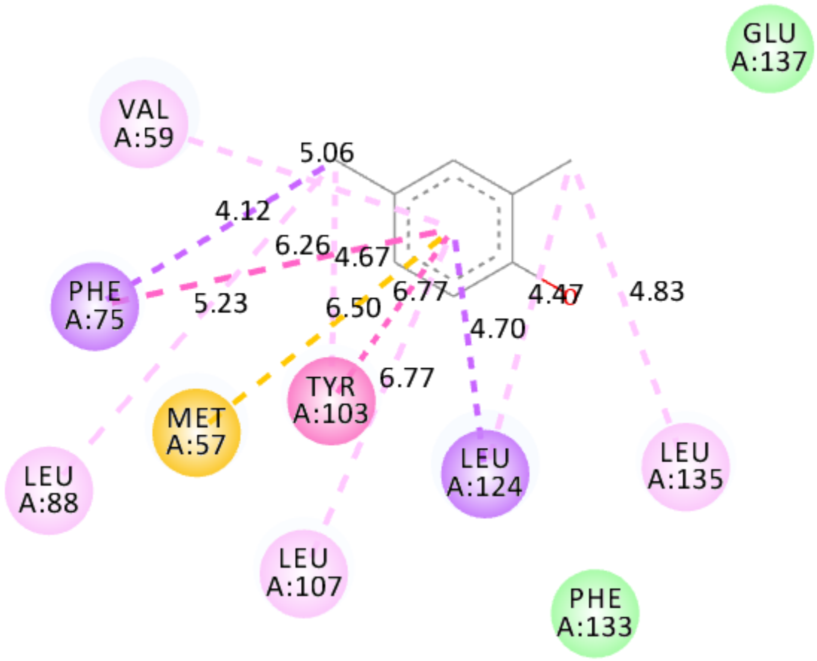

2D interaction pattern of 2,4-DMP.

L124V

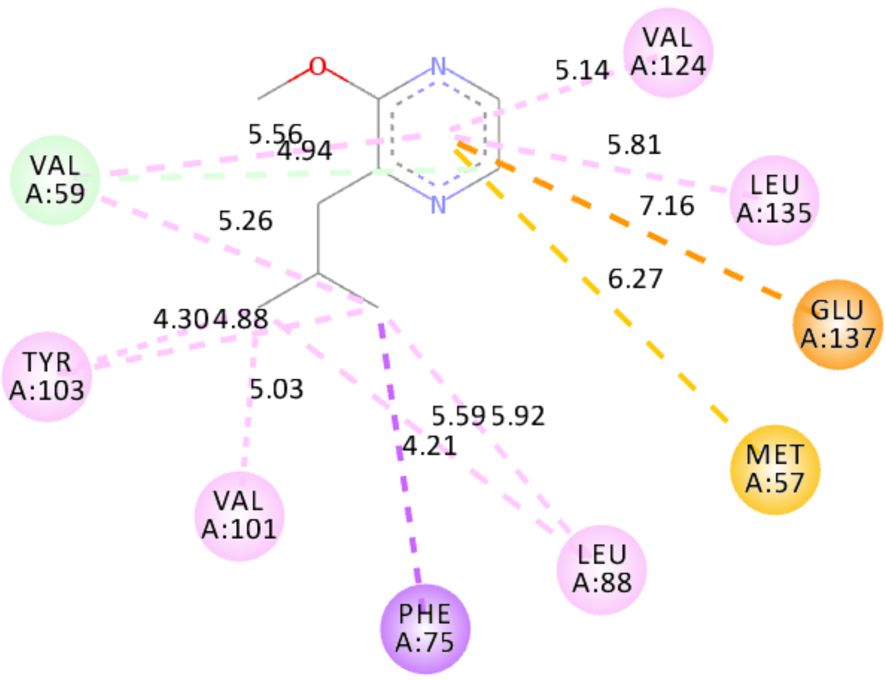

2D interaction pattern of PYR.

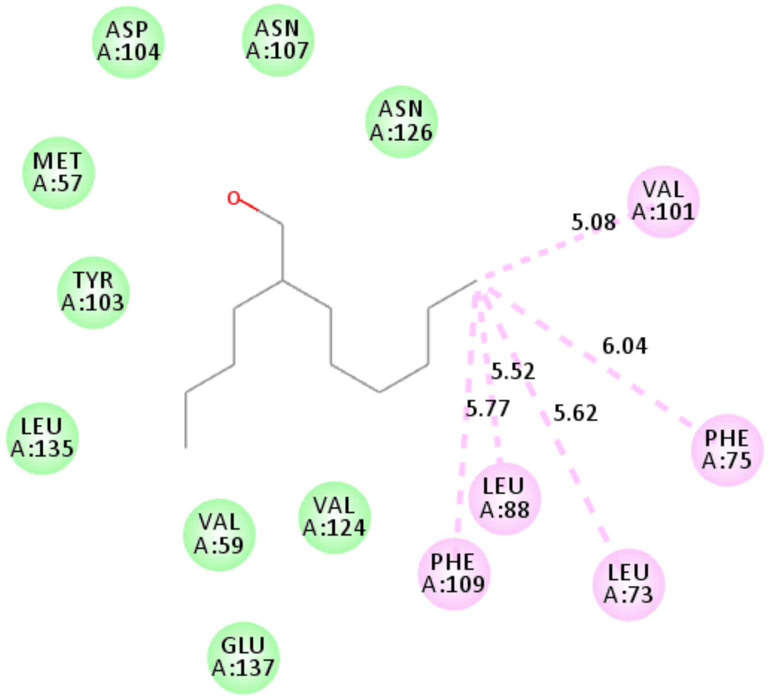

2D interaction pattern of OCT.

L124V

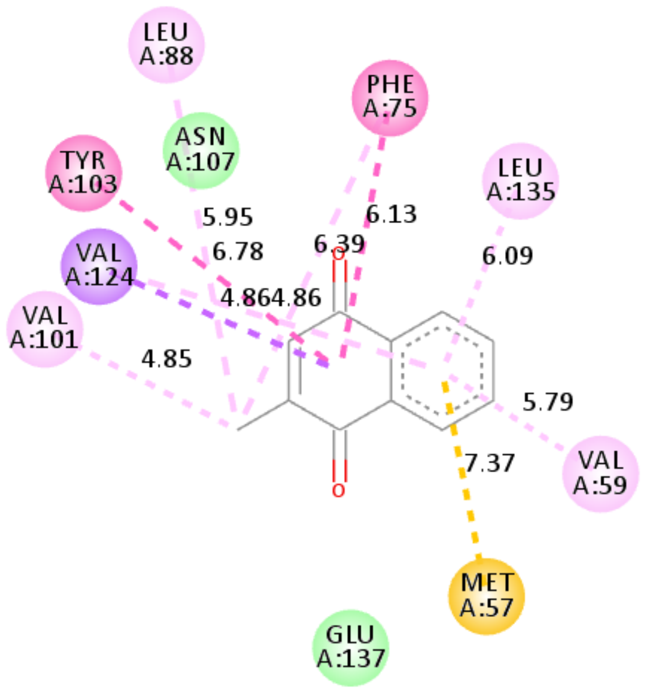

2D interaction pattern of MEN.

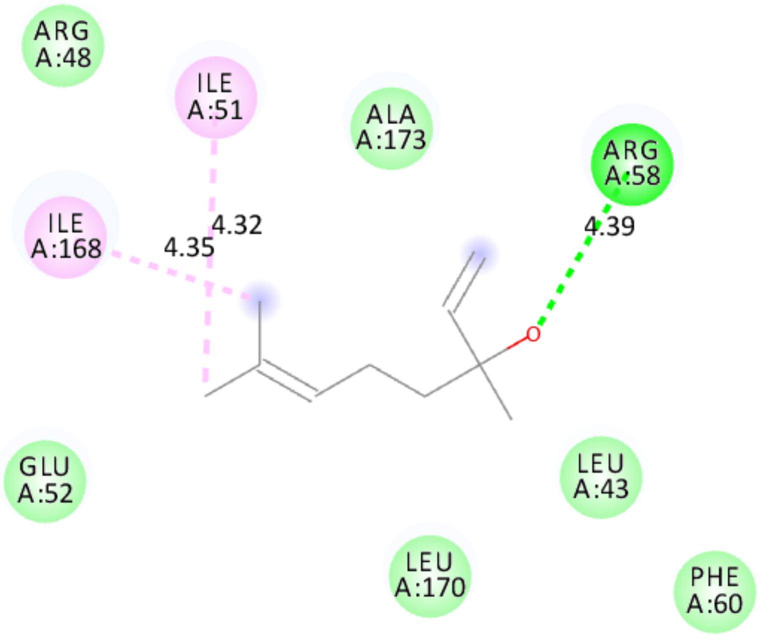

2D interaction pattern of LIN.

L124V

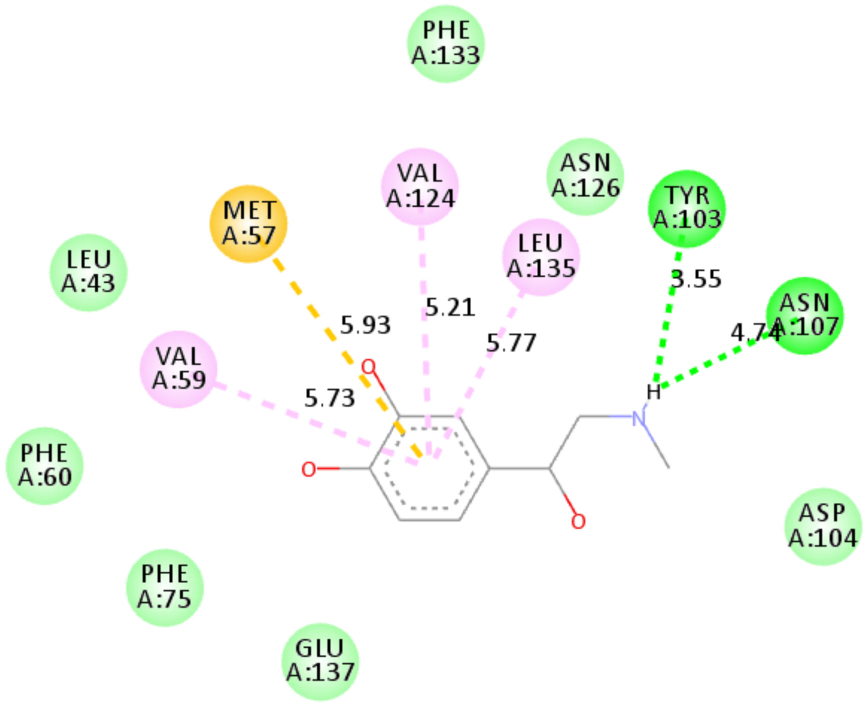

2D interaction pattern of L-ADR.

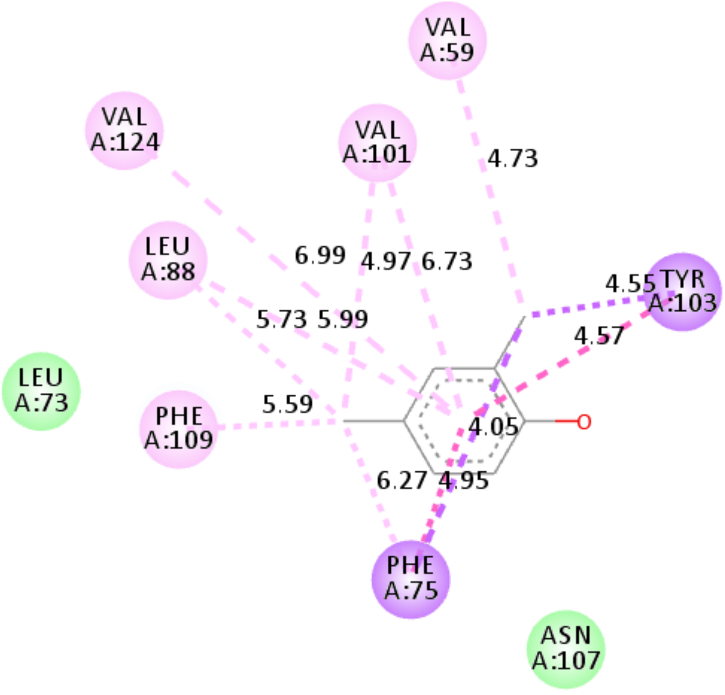

2D interaction pattern of 2,4-DMP.

E137K

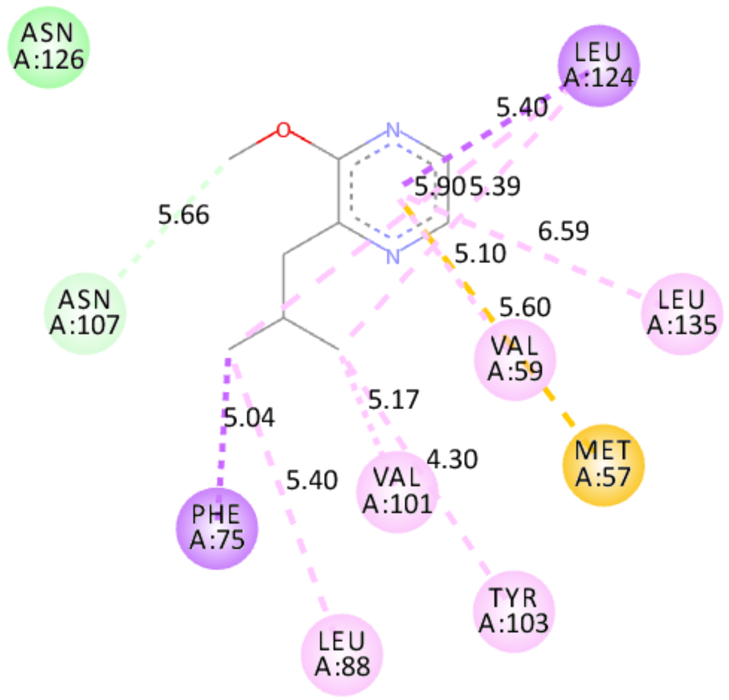

2D interaction pattern of PYR.

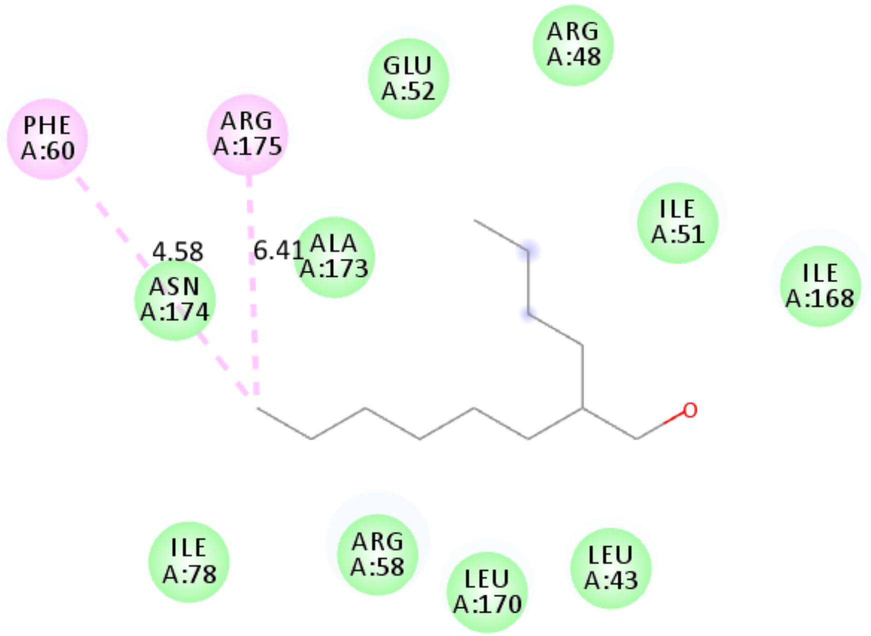

2D interaction pattern of OCT.

E137K

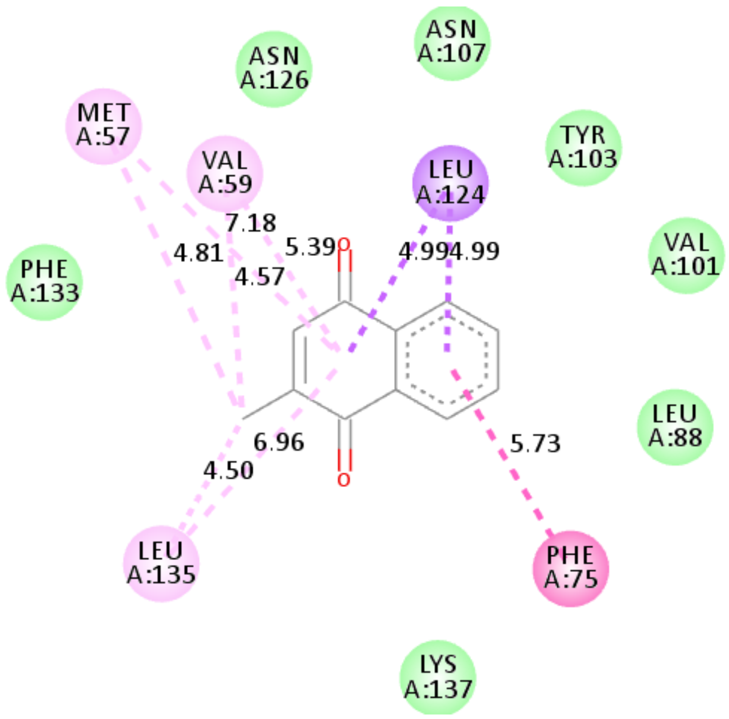

2D interaction pattern of MEN.

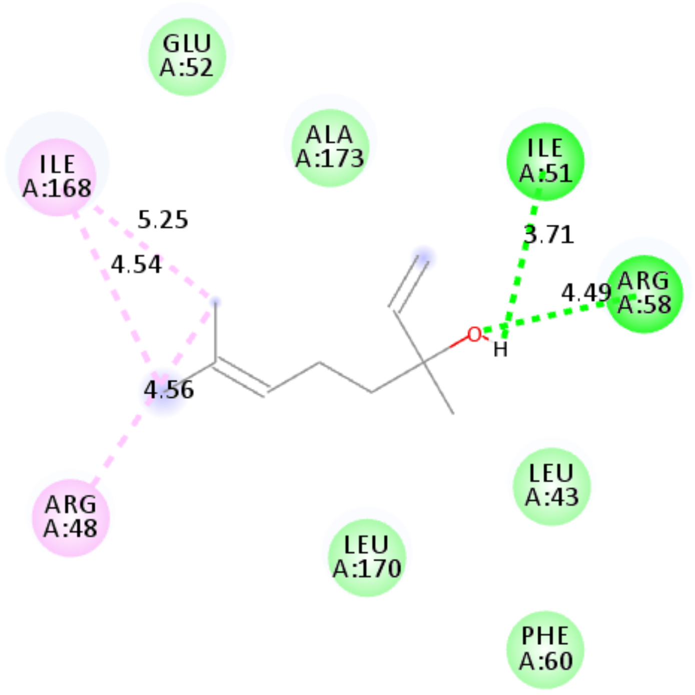

2D interaction pattern of LIN.

E137K

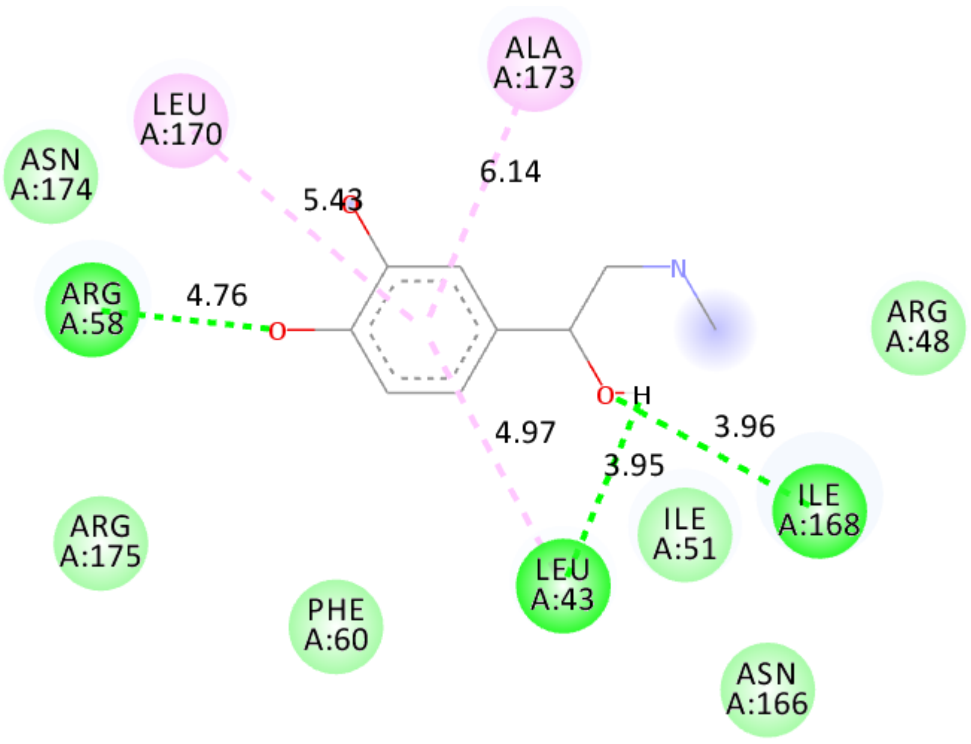

2D interaction pattern of L-ADR.

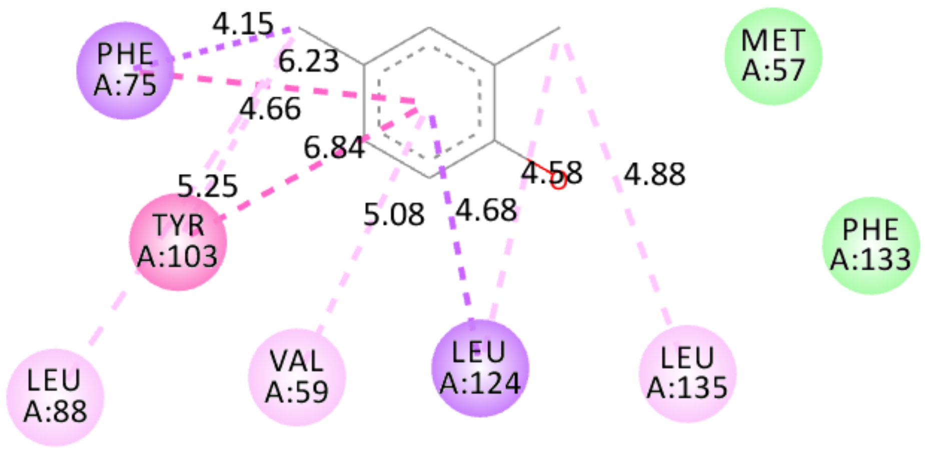

2D interaction pattern of 2,4-DMP.

Analysis of interacting residues

Protein	Ligand	Energy (-kcal/mol)	Interacting residues	Distance (Å)	Ligand-residue interaction
MUP20	OCT	4.7	Ile168	4.20	hydrophobic
	MEN	7.5	Met57	5.48	hydrophobic
			Val59	4.79	hydrophobic
			Leu124	3.37	π-σ
			Leu135	3.74	hydrophobic
			Phe75	5.04	π-π
	PYR	5.9	Asn107	3.37	van der Waals
			Val59	4.90	π-alkyl
			Leu124	3.82	π-σ
			Met57	5.20	π-sulfur
			Leu135	4.52	hydrophobic
			Tyr103	4.61	hydrophobic
			Val101	4.87	hydrophobic
			Leu88	4.66	hydrophobic
			Phe75	3.90	π-alkyl
	LIN	6.0	Val101	4.95	hydrophobic
			Leu124	4.65	hydrophobic
			Tyr103	4.52	hydrophobic
			Phe75	4.10	π-alkyl
			Leu88	4.07	hydrophobic
	L-ADR	5.7	Arg58	2.54	van der Waals
			Asn174	2.08	Hydrogen bond
			Leu43	2.56	Hydrogen bond
			Ile51	5.45	hydrophobic
			Ala173	4.79	hydrophobic
	2,4-DMP	6.4	Met57	4.78	hydrophobic
			Leu124	3.77	π-alkyl
			Val59	4.96	hydrophobic
			Tyr103	4.98	π-π
			Leu88	4.46	hydrophobic
			Phe75	4.23	π-π
V59T	OCT	5.2	Ile168	3.84	Hydrogen bond
			Leu43	4.13	Hydrogen bond
			Ile51	4.76	hydrophobic

	MEN	7.7	Met57	4.65	hydrophobic
			Leu124	5.19	π-σ
			Leu135	4.75	hydrophobic
	PYR	5.1	Leu43	4.42	hydrophobic
			Glu52	4.26	Hydrogen bond
			Ile51	5.29	π-alkyl
			Ala173	4.84	hydrophobic
			Leu170	4.46	hydrophobic
	LIN	5.0	Arg58	5.15	Hydrogen bond
			Ile51	4.75	hydrophobic
			Ile168	4.34	hydrophobic
			Arg48	4.77	hydrophobic
	L-ADR	5.4	Arg58	3.83	Hydrogen bond
			Ile51	3.37	Hydrogen bond
			Leu43	5.14	π-alkyl
	2,4-DMP	6.4	Thr59	3.65	Hydrogen bond
			Leu124	5.87	π-alkyl
			Val101	4.93	hydrophobic
			Tyr103	5.72	π-π
			Leu88	5.29	hydrophobic
			Phe75	4.51	π-alkyl
L88Q	OCT	5.0	Ile168	2.94	Hydrogen bond
			Leu43	4.21	Hydrogen bond
			Ile51	4.46	hydrophobic
	MEN	6.4	Leu43	4.46	hydrophobic
			Arg58	5.20	Hydrogen bond
			Leu170	4.34	hydrophobic
	PYR	5.1	Leu43	4.31	hydrophobic
			Ile51	5.29	π-alkyl
			Ala173	4.90	hydrophobic
			Leu170	4.72	hydrophobic
	LIN	5.8	Leu73	4.31	hydrophobic
			Val101	4.78	hydrophobic
			Tyr103	4.22	π-alkyl
	L-ADR	5.9	Tyr103	3.54	π-π
			Phe75	5.26	π-π

	2,4-DMP	6.3	Leu135	4.86	hydrophobic
			Leu124	4.71	π-alkyl
			Val59	4.68	hydrophobic
			Phe75	4.17	π-alkyl
Y103D	OCT	4.7	Ile168	3.68	Hydrogen bond
			Asn166	5.41	Hydrogen bond
	MEN	6.4	Ala173	5.50	π-alkyl
			Arg58	5.21	Hydrogen bond
			Leu170	4.33	hydrophobic
			Leu43	4.42	hydrophobic
	PYR	5.2	leu124	4.52	π-alkyl
			Leu135	5.25	hydrophobic
			Leu88	4.88	hydrophobic
			Phe75	3.91	π-alkyl
			Val59	4.30	van der Waals
	LIN	4.7	Leu73	3.09	Hydrogen bond
			Ile168	4.78	Hydrogen bond
			Ala173	4.88	hydrophobic
	L-ADR	5.2	Leu43	3.93	Hydrogen bond
			Ile168	3.79	Hydrogen bond
			Glu52	5.21	Hydrogen bond
			Ile51	5.25	Hydrogen bond
	2,4-DMP	5.6	Asn107	4.24	Hydrogen bond
			Leu124	5.14	π-alkyl
			Val59	4.82	hydrophobic
			Phe75	5.15	hydrophobic
Y03R	OCT	4.8	Arg103	3.19	Hydrogen bond
			Phe109	4.80	hydrophobic
	MEN	6.1	Leu71	5.06	π-alkyl
			Ile111	5.10	hydrophobic
	PYR	4.5	Ile111	4.93	π-alkyl
			Ile34	5.25	hydrophobic
			Ile64	5.11	hydrophobic
			Leu71	4.40	hydrophobic
	LIN	4.8	Ile111	4.28	hydrophobic
			Ile34	5.30	hydrophobic

	L-ADR	4.5	Ile111	5.26	Hphp
	2,4-DMP	4.9	Ile111	4.33	hydrophobic
			Ile122	4.37	hydrophobic
			Ile120	5.28	hydrophobic
			Ile64	5.03	hydrophobic
			Leu71	4.08	hydrophobic
			Leu43	4.89	hydrophobic
N107L	OCT	5.1	Ile168	3.99	Hydrogen bond
			Leu43	4.13	Hydrogen bond
			Arg58	3.93	hydrophobic
			Phe60	4.73	hydrophobic
	MEN	6.4	Arg58	5.20	Hydrogen bond
			Leu170	4.34	hydrophobicb
			Leu43	4.45	hydrophobic
	PYR	5.1	Glu52	4.22	Hydrogen bond
			Ile51	5.28	hydrophobic
			Ala173	4.85	hydrophobic
			Leu43	4.36	hydrophobic
			Leu170	4.70	hydrophobic
	LIN	5.8	Tyr103	4.62	π-alkyl
			Leu88	5.37	hydrophobic
			Phe109	4.70	hydrophobic
			Val101	4.50	hydrophobic
			Leu73	4.94	hydrophobic
			Phe75	3.94	π-alkyl
	L-ADR	5.3	Tyr103	5.40	π-π
			Val59	5.40	π-alkyl
			Leu124	5.49	π-alkyl
	2,4-DMP	6.5	Leu135	4.83	hydrophobic
			Leu124	4.70	π-alkyl
			Tyr103	4.67	π-alkyl
			Phe75	5.23	π-π
			Val59	5.06	hydrophobic
L124V	OCT	6.3	Val101	5.08	hydrophobic
	MEN	7.5	Val101	4.85	hydrophobic
			Val124	4.86	hydrophobic

			Tyr103	4.86	hydrophobic
	PYR	6.0	Val124	5.14	π-alkyl
			Phe75	4.21	π-alkyl
			Val101	5.03	hydrophobic
			Tyr103	4.30	hydrophobic
			Val59	4.94	π-alkyl
	LIN	5.2	Arg58	4.38	Hydrogen bond
			Ile168	4.35	hydrophobic
			Ile51	4.32	hydrophobic
	L-ADR	6.1	Tyr103	3.55	Hydrogen bond
			Asn107	4.74	Hydrogen bond
			Val124	5.21	π-alkyl
	2,4-DMP	6.1	Val59	4.73	hydrophobic
			Tyr103	4.57	π-π
			Phe75	4.95	π-π
			Val101	4.97	hydrophobic
E137K	OCT	5.3	Phe60	4.58	hydrophobic
	MEN	7.4	Leu124	4.99	π-alkyl
			Leu135	4.50	hydrophobic
			Met57	4.57	hydrophobic
	PYR	5.8	Leu124	5.40	π-alkyl
			Val59	5.10	hydrophobic
			Tyr103	4.30	hydrophobic
			Val101	5.17	hydrophobic
			Phe75	5.04	π-alkyl
	LIN	5.0	Ile51	3.71	Hydrogen bond
			Arg58	4.49	Hydrogen bond
			Arg48	4.56	hydrophobic
			Ile168	4.54	hydrophobic
	L-ADR	5.2	Ile168	3.96	Hydrogen bond
			Leu43	3.95	Hydrogen bond
			Arg58	4.76	Hydrogen bond
			Leu170	5.43	hydrophobic
	2,4-DMP	6.2	Leu135	4.88	hydrophobic
			Leu124	4.68	π-alkyl
			Tyr103	5.25	π-alkyl
			Phe75	4.15	π-alkyl

Supplementary Material S2

S2a. List of selected aminoacids for mutation and mutation scheme

V59, L88, Y103, N107, L124, E137.

Mutation and alignment Scheme

7 mutated proteins: MUP20V59T, MUP20L88Q, MUP20Y103D, MUP20Y103R, MUP20N107L, MUP20L124V, MUP20E137K.

```
CLUSTAL format alignment by MAFFT (v7.243)


MUP20wt         M----HHHHHHMKLLVLLLCLGLTLVCVHAEEASSMERNFNVEKINGEWYTIMLATDKRE
MUP20V59T       MMGSSHHHHHHIE---------------GREEASSMERNFNVEKINGEWYTIMLATDKRE
MUP20L124V      MMGSSHHHHHHIE---------------GREEASSMERNFNVEKINGEWYTIMLATDKRE
MUP20E137K      MMGSSHHHHHHIE---------------GREEASSMERNFNVEKINGEWYTIMLATDKRE
MUP20L88Q       MMGSSHHHHHHIE---------------GREEASSMERNFNVEKINGEWYTIMLATDKRE
MUP20N107L      MMGSSHHHHHHIE---------------GREEASSMERNFNVEKINGEWYTIMLATDKRE
MUP20Y103R      MMGSSHHHHHHIE---------------GREEASSMERNFNVEKINGEWYTIMLATDKRE
MUP20Y103D      MMGSSHHHHHHIE---------------GREEASSMERNFNVEKINGEWYTIMLATDKRE
                *    ******::                 ******************************

MUP20wt         KIEEHGSMRVFVEYIHVLENSLALKFHIIINEECSEIFLVADKTEKAGEYSVTYDGSNTF
MUP20V59T       KIEEHGSMRTFVEYIHVLENSLALKFHIIINEECSEIFLVADKTEKAGEYSVTYDGSNTF
MUP20L124V      KIEEHGSMRVFVEYIHVLENSLALKFHIIINEECSEIFLVADKTEKAGEYSVTYDGSNTF
MUP20E137K      KIEEHGSMRVFVEYIHVLENSLALKFHIIINEECSEIFLVADKTEKAGEYSVTYDGSNTF
MUP20L88Q       KIEEHGSMRVFVEYIHVLENSLALKFHIIINEECSEIFQVADKTEKAGEYSVTYDGSNTF
MUP20N107L      KIEEHGSMRVFVEYIHVLENSLALKFHIIINEECSEIFLVADKTEKAGEYSVTYDGSNTF
MUP20Y103R      KIEEHGSMRVFVEYIHVLENSLALKFHIIINEECSEIFLVADKTEKAGEYSVTRDGSNTF
MUP20Y103D      KIEEHGSMRVFVEYIHVLENSLALKFHIIINEECSEIFLVADKTEKAGEYSVTDDGSNTF
                *********.**************************** ************** ******

MUP20wt         TILKTDYDNYIMIHLINKKDGETFQLMELYGREPDLSSDIKEKFAQLSEEHGIVRENIID
MUP20V59T       TILKTDYDNYIMIHLINKKDGETFQLMELYGREPDLSSDIKEKFAQLSEEHGIVRENIID
MUP20L124V      TILKTDYDNYIMIHVINKKDGETFQLMELYGREPDLSSDIKEKFAQLSEEHGIVRENIID
MUP20E137K      TILKTDYDNYIMIHLINKKDGETFQLMKLYGREPDLSSDIKEKFAQLSEEHGIVRENIID
MUP20L88Q       TILKTDYDNYIMIHLINKKDGETFQLMELYGREPDLSSDIKEKFAQLSEEHGIVRENIID
MUP20N107L      TILKTDYDLYIMIHLINKKDGETFQLMELYGREPDLSSDIKEKFAQLSEEHGIVRENIID
MUP20Y103R      TILKTDYDNYIMIHLINKKDGETFQLMELYGREPDLSSDIKEKFAQLSEEHGIVRENIID
MUP20Y103D      TILKTDYDNYIMIHLINKKDGETFQLMELYGREPDLSSDIKEKFAQLSEEHGIVRENIID
                ******** *****:************:********************************

MUP20wt         LTNANRCLEARE
MUP20V59T       LTNANRCLEARE
MUP20L124V      LTNANRCLEARE
MUP20E137K      LTNANRCLEARE
MUP20L88Q       LTNANRCLEARE
MUP20N107L      LTNANRCLEARE
MUP20Y103R      LTNANRCLEARE
MUP20Y103D      LTNANRCLEARE
                ************
```

Figure S2a Clustal alignment of amino acid sequences

S2b. Expression Procedure

For the heterologous expression 100µl of DE3 BL21 *E. coli* cells strains were used, after the preparation of competent bacteria, followed by a heat shock transformation with 10µl of ligation product containing the plasmid including the ampicillin resistance gene. Transformants carrying the plasmid for wild-type or mutant MUP20 were plated in LB-agar 100µg/ml ampicillin Petri dishes and incubated overnight at 37°C. In order to proceed with the small scale expression of the recombinant proteins, a set of single antibiotic resistant colony was selected to inoculate 5 ml of ampicillin-LB media and incubated with shaking 250 rpm at 37°C. The turbid liquid cultures (positives) were selected for the bacterial growing step.

From each liquid culture 750 µl were mixed with 75 ml of ampicillin-LB media, shaken at 300 rpm at 37°C until the OD600 reached 0.4-0.6. Subsequently, for transcription of the gene of interest, the protein expression was induced by isopropyl-b-D-thiogalactoside (0.4mM, BioChemica, EuroClone, Milan, Italy) and shaken at 350 rpm at 37°C for 3 hours. The liquid culture was centrifuged at 5000 rpm for 10', and pellets were collected and sampled to confirm the presence of the protein via SDS-page.

Figure S1b shows an example of MUP expression, SDS-PAGE (15% acrylamide) analysis of recombinant MUP under reducing conditions. Lane 1 depicts MUP20 L88Q before induction and lane 2 after induction. Lane 3 shows MUP20 L124V before induction and lane 4 after induction. On the right, molecular weight markers are shown. The original gel is shown in Figure S1c.

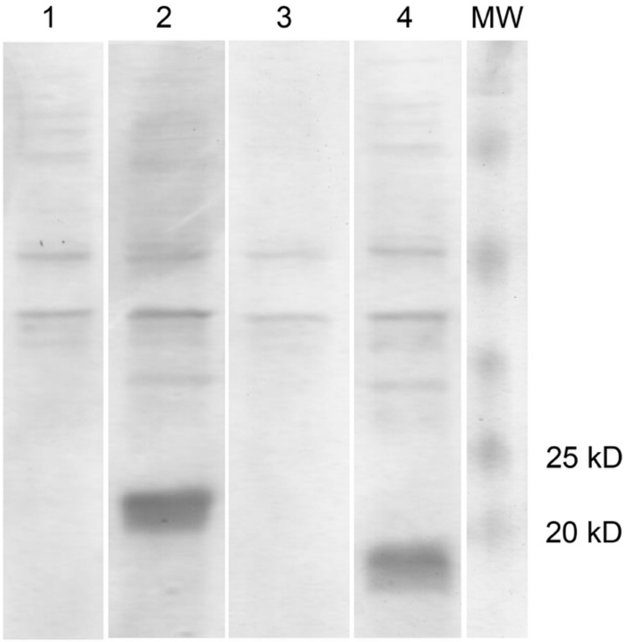

Figure S2b SDS-PAGE of recombinant MUPs

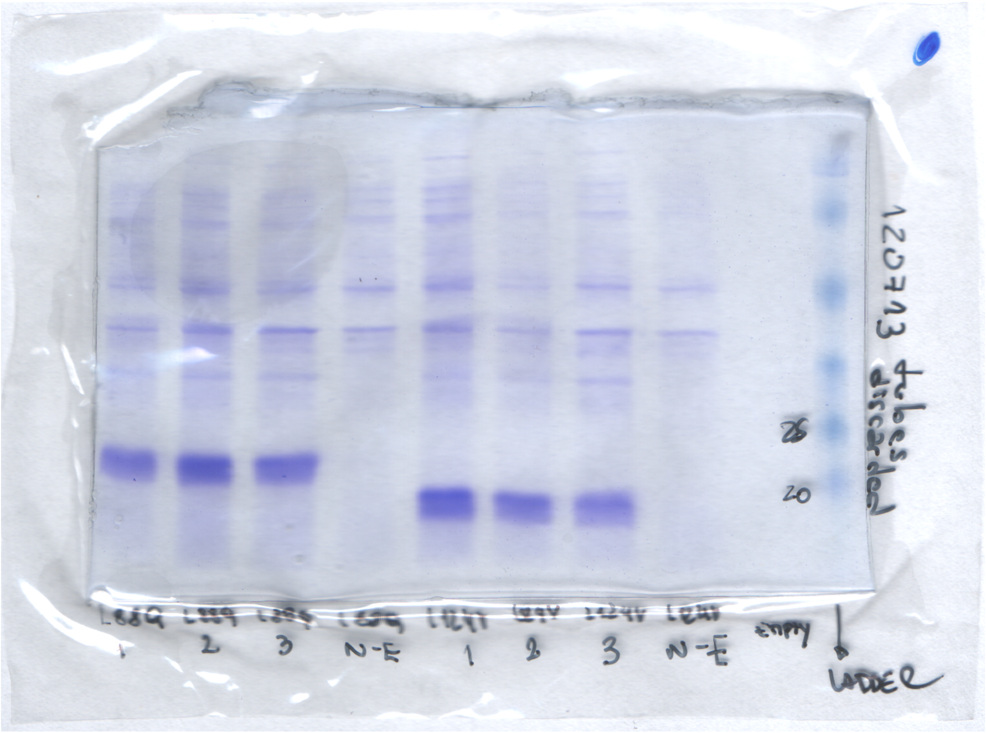

Figure S2c. Unmodified scanning of the gel showed in Fig. S1b.

Supplementary Material S 3

Measured molecular weights from Mass Spectrometry and theoretical values

MUP	MW (experimental) – a.m.u	MW (theoretical) – a.m.u.
WT	20625.2	21883.97
L88Q	20621.4	20682.16
L124V	20653.5	20653.16
Y103R	20628.3	20660.20

Supplementary Material S4. Measured Kd (μM)

	WT		V59T		L88Q		Y103D		Y103R		N107L		L124V		E137K	
**Ligand**	**Kd**	**SD**	**Kd**	**SD**	**Kd**	**SD**	**Kd**	**SD**	**Kd**	**SD**	**Kd**	**SD**	**Kd**	**SD**	**Kd**	**SD**
**PYR**	1.29	0.01	1.93	0.00	1.39	0.01	0.84	0.04	0.74	0.00	1.40	0.01	0.07	0.01	1.52	0.02
**LIN**	2.82	0.04	1.60	0.00	2.21	0.01	2.17	0.06	5.93	0.11	1.37	0.02	0.29	0.01	1.85	0.05
**2,4-DMP**	4.24	0.07	1.44	0.02	5.08	0.29	4.68	0.09	11.92	0.09	1.99	0.03	1.30	0.06	4.05	0.04
**OCT**	0.78	0.00	1.54	0.02	1.45	0.03	0.92	0.01	1.88	0.01	2.67	0.08	0.36	0.00	3.20	0.01
**MEN**	0.61*	0.04	1.85	0.06	1.31*	0.06	0.82*	0.10	1.33*	0.06	1.23	0.05	0.07*	0.01	2.14	0.29
**L-ADR**	5.31	0.06	0.87	0.01	11.24	0.58	3.34	0.25	7.06	0.23	0.90	0.03	1.33	0.06	4.07	0.45

*data from Scorsone et al., IEEE Sensors Journal 2016, 16, 6543, Tab. 1.

Supplementary Material S5. Binding data

	**MUP20x6HisWT**	first	second				first	second				
	Ligand	Kd	Kd	**Kd mean**	**Kd SD**	Ligand	Ka - 1	Ka - 2	**Ka mean**	**Ka SD**	=(IC-50/(1+1-NPNfree/1-NPNkd)	
1.00 uM	2-isobutyl-3-methoxypyrazine	1.29	1.28	**1.29**	**0.01**	2-isobutyl-3-methoxypyrazine	0.77	0.78	**0.78**	**0.01**		
1.60 uM	Linalool	2.85	2.80	**2.82**	**0.04**	Linalool	0.35	0.36	**0.35**	**0.01**	Kd 1-NPN	
	2.4-dimethylphenol	4.20	4.29	**4.24**	**0.07**	2.4-dimethylphenol	0.24	0.23	**0.24**	**0.00**		0.65
	2-butyl-1-octanol	0.78	0.78	**0.78**	**0.00**	2-butyl-1-octanol	1.28	1.28	**1.28**	**0.00**	1+1-NPNfree/Kd	
	MEN	0.64	0.59	**0.61**	**0.04**	MEN	1.56	1.71	**1.63**	**0.10**		2.43
	L-ADR	5.36	5.27	**5.31**	**0.06**	L-ADR	0.19	0.19	**0.19**	**0.00**		
	**MUP20x6HisV59T**											
	Ligand	Kd	Kd	**Kd mean**	**Kd SD**	Ligand	Ka - 1	Ka - 2	**Ka mean**	**Ka SD**	=(IC-50/(1+1-NPNfree/1-NPNkd)	
1.00 uM	2-isobutyl-3-methoxypyrazine	1.93	1.94	**1.93**	**0.00**	2-isobutyl-3-methoxypyrazine	0.52	0.52	**0.52**	**0.00**		
1.60 uM	Linalool	1.60	1.60	**1.60**	**0.00**	Linalool	0.63	0.62	**0.62**	**0.00**	Kd 1-NPN	
	2.4-dimethylphenol	1.42	1.45	**1.44**	**0.02**	2.4-dimethylphenol	0.70	0.69	**0.70**	**0.01**		0.46
	2-butyl-1-octanol	1.55	1.52	**1.54**	**0.02**	2-butyl-1-octanol	0.64	0.66	**0.65**	**0.01**	1+1-NPNfree/Kd	
	Menadione	1.89	1.80	**1.85**	**0.06**	Menadione	0.53	0.55	**0.54**	**0.02**		3.09
	L-Adrenaline	0.86	0.88	**0.87**	**0.01**	L-Adrenaline	1.16	1.14	**1.15**	**0.02**		
	**MUP20x6HisL88Q**											
	Ligand	Kd	Kd	**Kd mean**	**Kd SD**	Ligand	Ka - 1	Ka - 2	**Ka mean**	**Ka SD**	=(IC-50/(1+1-NPNfree/1-NPNkd)	
1.00 uM	2-isobutyl-3-methoxypyrazine	1.39	1.40	**1.39**	**0.01**	2-isobutyl-3-methoxypyrazine	0.72	0.71	**0.72**	**0.00**		
1.60 uM	Linalool	2.21	2.20	**2.21**	**0.01**	Linalool	0.45	0.45	**0.45**	**0.00**	Kd 1-NPN	
	2.4-dimethylphenol	5.28	4.88	**5.08**	**0.29**	2.4-dimethylphenol	0.19	0.21	**0.20**	**0.01**		2.07
	2-butyl-1-octanol	1.48	1.43	**1.45**	**0.03**	2-butyl-1-octanol	0.68	0.70	**0.69**	**0.02**	1+1-NPNfree/Kd	
	Menadione	1.27	1.36	**1.31**	**0.06**	Menadione	0.79	0.74	**0.76**	**0.04**		1.46
	L-Adrenaline	11.65	10.83	**11.24**	**0.58**	L-Adrenaline	0.09	0.09	**0.09**	**0.00**		
	**MUP20x6HisY103D**											
	Ligand	Kd	Kd	**Kd mean**	**Kd SD**	Ligand	Ka - 1	Ka - 2	**Ka mean**	**Ka SD**	=(IC-50/(1+1-NPNfree/1-NPNkd)	
1.00 uM	2-isobutyl-3-methoxypyrazine	0.87	0.82	**0.84**	**0.04**	2-isobutyl-3-methoxypyrazine	1.15	1.22	**1.18**	**0.05**		
1.60 uM	Linalool	2.21	2.13	**2.17**	**0.06**	Linalool	0.45	0.47	**0.46**	**0.01**	Kd 1-NPN	
	2.4-dimethylphenol	4.61	4.74	**4.68**	**0.09**	2.4-dimethylphenol	0.22	0.21	**0.21**	**0.00**		1.57
	2-butyl-1-octanol	0.91	0.92	**0.92**	**0.01**	2-butyl-1-octanol	1.09	1.08	**1.09**	**0.01**	1+1-NPNfree/Kd	
	Menadione	0.75	0.89	**0.82**	**0.10**	Menadione	1.32	1.12	**1.22**	**0.15**		1.55
	L-Adrenaline	3.17	3.52	**3.34**	**0.25**	L-Adrenaline	0.32	0.28	**0.30**	**0.02**		
	**MUP20x6HisY103R**											
	Ligand	Kd	Kd	**Kd mean**	**Kd SD**	Ligand	Ka - 1	Ka - 2	**Ka mean**	**Ka SD**	=(IC-50/(1+1-NPNfree/1-NPNkd)	
1.00 uM	2-isobutyl-3-methoxypyrazine	0.74	0.74	**0.74**	**0.00**	2-isobutyl-3-methoxypyrazine	1.36	1.36	**1.36**	**0.00**		
1.60 uM	Linalool	5.86	6.01	**5.93**	**0.11**	Linalool	0.17	0.17	**0.17**	**0.00**	Kd 1-NPN	
	2.4-dimethylphenol	11.86	11.98	**11.92**	**0.09**	2.4-dimethylphenol	0.08	0.08	**0.08**	**0.00**		4.39
	2-butyl-1-octanol	1.88	1.88	**1.88**	**0.01**	2-butyl-1-octanol	0.53	0.53	**0.53**	**0.00**	1+1-NPNfree/Kd	
	Menadione	1.37	1.29	**1.33**	**0.06**	Menadione	0.73	0.77	**0.75**	**0.03**		1.21
	L-Adrenaline	7.22	6.90	**7.06**	**0.23**	L-Adrenaline	0.14	0.14	**0.14**	**0.00**		
	**MUP20x6HisN107L**											
	Ligand	Kd	Kd	**Kd mean**	**Kd SD**	Ligand	Ka - 1	Ka - 2	**Ka mean**	**Ka SD**	=(IC-50/(1+1-NPNfree/1-NPNkd)	
1,00 uM	2-isobutyl-3-methoxypyrazine	1.40	1.39	**1.40**	**0.01**	2-isobutyl-3-methoxypyrazine	0.71	0.72	**0.72**	**0.00**		
1,60 uM	Linalool	1.39	1.36	**1.37**	**0.02**	Linalool	0.72	0.74	**0.73**	**0.01**	Kd 1-NPN	
	2.4-dimethylphenol	1.97	2.01	**1.99**	**0.03**	2.4-dimethylphenol	0.51	0.50	**0.50**	**0.01**		0.85
	2-butyl-1-octanol	2.73	2.62	**2.67**	**0.08**	2-butyl-1-octanol	0.37	0.38	**0.37**	**0.01**	1+1-NPNfree/Kd	
	Menadione	1.19	1.27	**1.23**	**0.05**	Menadione	0.84	0.79	**0.81**	**0.03**		2.05
	L-Adrenaline	0.92	0.88	**0.90**	**0.03**	L-Adrenaline	1.09	1.14	**1.11**	**0.03**		
	**MUP20x6HisL124V**											
	Ligand	Kd	Kd	**Kd mean**	**Kd SD**	Ligand	Ka - 1	Ka - 2	**Ka mean**	**Ka SD**	=(IC-50/(1+1-NPNfree/1-NPNkd)	
1.00 uM	2-isobutyl-3-methoxypyrazine	0.06	0.07	**0.07**	**0.01**	2-isobutyl-3-methoxypyrazine	17.00	13.42	**15.21**	**2.53**		
1.60 uM	Linalool	0.30	0.29	**0.29**	**0.01**	Linalool	3.34	3.46	**3.40**	**0.09**	Kd 1-NPN	
	2.4-dimethylphenol	1.34	1.26	**1.30**	**0.06**	2.4-dimethylphenol	0.74	0.79	**0.77**	**0.03**		0.22
	2-butyl-1-octanol	0.36	0.36	**0.36**	**0.00**	2-butyl-1-octanol	2.79	2.76	**2.78**	**0.02**	1+1-NPNfree/Kd	
	Menadione	0.06	0.07	**0.07**	**0.01**	Menadione	17.49	13.34	**15.42**	**2.93**		5.03
	L-Adrenaline	1.29	1.37	**1.33**	**0.06**	L-Adrenaline	0.78	0.73	**0.75**	**0.03**		
	**MUP20x6HisE137K**											
	Ligand	Kd	Kd	**Kd mean**	**Kd SD**	Ligand	Ka - 1	Ka - 2	**Ka mean**	**Ka SD**	=(IC-50/(1+1-NPNfree/1-NPNkd)	
1.00 uM	2-isobutyl-3-methoxypyrazine	1.51	1.54	**1.52**	**0.02**	2-isobutyl-3-methoxypyrazine	0.66	0.65	**0.66**	**0.01**		
1.60 uM	Linalool	1.82	1.89	**1.85**	**0.05**	Linalool	0.55	0.53	**0.54**	**0.01**	Kd 1-NPN	
	2.4-dimethylphenol	4.02	4.07	**4.05**	**0.04**	2.4-dimethylphenol	0.25	0.25	**0.25**	**0.00**		1.36
	2-butyl-1-octanol	3.19	3.20	**3.20**	**0.01**	2-butyl-1-octanol	0.31	0.31	**0.31**	**0.00**	1+1-NPNfree/Kd	
	Menadione	1.93	2.34	**2.14**	**0.29**	Menadione	0.52	0.43	**0.47**	**0.06**		1.65
	L-Adrenaline	3.75	4.39	**4.07**	**0.45**	L-Adrenaline	0.27	0.23	**0.25**	**0.03**		
